# Prediction and validation of the structural features of Ov58GPCR, an immunogenic determinant of *Onchocerca volvulus*

**DOI:** 10.1371/journal.pone.0202915

**Published:** 2018-09-26

**Authors:** Robert Adamu Shey, Stephen Mbigha Ghogomu, Ferdinand Ngale Njume, Lea Olive Tchouate Gainkam, Philippe Poelvoorde, Leon Mutesa, Annie Robert, Perrine Humblet, Jean-Pierre Munyampundu, Joseph Kamgno, Christophe Lelubre, Luc Vanhamme, Jacob Souopgui

**Affiliations:** 1 Department of Molecular Biology, Institute of Biology and Molecular Medicine, IBMM Universite Libre de Bruxelles, Gosselies Campus, Gosselies, Hainaut, Belgium; 2 Molecular and Cell Biology Laboratory, Biotechnology Unit, Faculty of Science, University of Buea, Buea, Cameroon; 3 Center for Human Genetics, College of Medicine and Health Sciences, University of Rwanda, Kigali, Rwanda; 4 Faculté de santé publique, Institut de recherche expérimentale et clinique, Pôle d'épidémiologie et biostatistique, Université Catholique de Louvain, Clos Chapelle-aux-champs, Woluwe-Saint-Lambert, Belgium; 5 École de santé publique, Université Libre de Bruxelles, Bruxelles, Belgium; 6 Department of Epidemiology, Centre for research on filariasis and other tropical diseases, (CRFilMT), Yaoundé, Cameroon; 7 Laboratoire de Médecine Expérimentale, Université Libre de Bruxelles (ULB) - Unité 222, CHU Charleroi (Hôpital André Vésale), Montigny-Le-Tilleul, Belgium; Centers for Disease Control and Prevention, UNITED STATES

## Abstract

Onchocerciasis is a severely debilitating yet neglected tropical disease (NTD) that creates social stigma, generates and perpetuates poverty, and leads ultimately in some cases to irreversible unilateral or bilateral blindness if untreated. Consequently, the disease is a major impediment to socioeconomic development. Many control programs have been launched for the disease with moderate successes achieved. This mitigated hit is partially due to the lingering need for reliable, non-invasive and easily applicable tools for mapping endemic regions and post-elimination surveillance. In this work, bioinformatics analyses combined with immunological assays were applied in a bid to develop potential tools for diagnosis and assessing the success of drug treatment programs. We report that (i) the *O*. *volvulus* antigen, Ov58GPCR is a G-protein coupled receptor (GPCR) conserved in related nematodes, (ii) synthetic peptides predicted to be in the extracellular domain (ECD) of Ov58GPCR are indeed immunogenic epitopes in actively-infected individuals, (iii) synthetic peptide cocktails discriminate between actively-infected individuals, treated individuals and healthy African controls, (iv) polyclonal antibodies against one of the peptides or against the bacterially-expressed ECD reacted specifically with the native antigen of *O*. *volvulus* total and surface extracts, (v) Ov58GPCR is transcribed in both larvae and adult parasite stages, (vi) IgG and IgE responses to the recombinant ECD decline with ivermectin treatment. All these findings suggest that the extracellular domain and synthetic peptides of Ov58GPCR, as well as the specific immune response generated could be harnessed in the context of disease diagnosis and surveillance.

## Introduction

River blindness (onchocerciasis) is a neglected tropical disease (NTD) which results in severe itching, skin lesions and diverse degrees of vision impairment [1]. The disease, caused by the nematode parasite *Onchocerca volvulus* is transmitted by repeated bites from infected blackflies of the genus *Simulium* [2]. It is the second leading infectious cause of blindness worldwide [3] and currently affects approximately 15.5 million people including 12.2 million with onchocerca skin disease (OSD) and 1.025 million with vision loss (river blindness). Most people affected with OSD and river blindness live in the rural areas in sub-Saharan Africa or Yemen [4]. An additional 172 million people are reported to need preventive chemotherapy [5]. Furthermore, though *O*. *volvulus* infection has been regarded as the cause of a chronic yet non-fatal condition, recent analyses have indicated that in addition to blindness, the parasite is also directly associated with human mortality [6], blindness giving rise to a significant increase in mortality [7]. The enormity of its public health and socio-economic burden stimulated the creation of various large scale control programmes [8] the first of which was initiated as far back as 1974 [9].

Control programmes have achieved some successes: eliminating the disease in 4 of 6 endemic countries in the Americas and in selected foci in Africa [10–13]. These successes have led to the shift in treatment goals of onchocerciasis from control of morbidity to elimination [14]. The World Health Organisation (WHO), through the Expanded Special Project for Elimination of Neglected Tropical Diseases (ESPEN) is working towards achieving onchocerciasis elimination in selected African countries by 2020 and in at least 80% of African countries by 2025 [5,15]. However, drug resistance, drug contra-indication in cases of co-endemicity with loiasis, recrudescence and potential on-going transmission in hypo-endemic areas in between others remain a threat to onchocerciasis elimination in Africa [16–20]. The ultimate achievement of WHO’s goal is in need of a wide range of novel tools: amongst others, tools for specific diagnosis to accurately map endemic areas [21], through chemotherapeutics (both macrofilaricides and microfilaricides) [22] to tools that can certify the elimination of the disease in endemic zones and also help to monitor recrudescence [21,23].

Currently, the Ov16 ELISA is the only antibody-detection system used in onchocerciasis field diagnosis and for assessment of the interruption of transmission of *O*. *volvulus* [24,25]. It has however been reported that not all those with patent onchocerciasis infection develop antibodies against Ov16. It has therefore been suggested that a single antibody test is not fully adequate for epidemiological surveillance purposes [26]. Also, no field studies have been recently published on the performance of the Ov16 test in different endemic settings [21]. These limitations indicate that there is a need for a continuous search of antigens that could be used in onchocerciasis control as tools for diagnosis, monitoring success of ivermectin treatment programs and determination of treatment endpoints.

A critical step in the development of control tools is the identification and characterisation of specific molecules from the parasite [27]. Excellent candidate molecules to develop such tools are those directly exposed to the host’s immune system such as excretory secretory products (ESPs) or cuticular surface products of the parasites [28]. To circumvent cross-reactivity due to sequence identity amongst different species’ orthologous proteins, epitopes or specific non-conserved immunogenic portions of the molecules of interest should be used [29–31]. *In silico* predictions are therefore helpful to identify domains and epitopes.

Synthetic peptides have been successfully used to improve diagnosis (achieving very high specificities and sensitivities) of many infectious diseases including schistosomiasis [32], visceral leishmaniasis [33] or trypanosomiasis [34]. The increasing numbers of sequenced nematode genomes and proteomes [35–38] and the large numbers of open source bioinformatics tools and webservers available has eased the *in-silico* characterisation of some of these molecules and aided to streamline those that can be further characterised [39].

The protein Ov-47, (UniProt ID:O46146) was reported to be a dominant antigen of *O*. *volvulus* adult worms with potential role in the host–parasite interplay [27]. The *Onchocerca volvulus* protein OVOC5284 (renamed Ov58GPCR) was confirmed to be the expressed full-length sequence of Ov-47. In this communication we further characterised this protein classified as a G-protein coupled receptor (GPCR). The extracellular domains of GPCRs have been reported to play critical roles in GPCR signalling, being involved in oligomerisation, ligand binding, receptor activation and regulation of signalling [40,41]. In an attempt to characterise Ov58GPCR in the context of onchocerciasis control, the extracellular domain (ECD) and some of its linear B-epitopes were predicted and validated as immunogenic determinants. Both the synthetic peptides and Ov58GPCR-ECD demonstrated characteristics related to our study goals.

## Materials and methods

### Study design and subject group composition

The objective of this study was to assess the potential role of the *Onchocerca volvulus* antigen Ov58GPCR in the development of tools to be used in onchocerciasis control in the areas of diagnosis and in monitoring the success of drug treatment programs. To achieve this, serum samples from six subject groups consisting of infected individuals and various controls were composed.

The first group consisting of individuals actively-infected with *Onchocerca voluvulus* (OVS—Onchocerca volvulus serum) was recruited in communities from Malende and Kombone health areas in the health districts (HDs) of Muyuka and Mbonge respectively, in the South West Region of Cameroon. This region was reported in 2006 to be hyperendemic for onchocerciasis [42]. Sample collection method here was random cross-sectional. Apparently healthy persons aged 5 years and above who presented at the screening centres (both with or without any clinical symptoms of onchocerciasis) were recruited in the study.

The endemic normal (putatively resistant or immune) group designated Endemic Non-Symptomatic (ENS) consisted of persons who were born and raised or have lived and worked in the endemic regions of the Mbonge and Muyuka health districts earlier mentioned for extended periods without any cutaneous or ocular signs and symptoms of the disease. This group consisted of ivermectin naïve-individuals who proved microfilaria-negative following skin-snip microscopy. Samples for this ENS group and the actively-infected (OVS) group were collected between November 2015 and June 2016 following examination by trained medical personnel for ocular and cutaneous lesions. Apparently healthy persons aged 5 and above who presented at the screening centres (both with or without any clinical symptoms of onchocerciasis) were recruited in the study.

The group from an onchocerciasis hypo-endemic (HES—hypo-endemic serum) setting consisted of randomly selected apparently healthy residents in the Huye district in the Southern province of Rwanda. Rwanda is considered a low risk zone for onchocerciasis, [43]. Serum samples from this population were collected in October 2017.

The group from the onchocerciasis-near elimination region (ITS—ivermectin treatment serum) consisted of a targeted population of males and females aged least 5 years from 3 communities (Tsesse, Lemgo and Se Dembom) in the Bandjoun Health District in the West Region of Cameroon previously tested in May 2015 by skin-snip microscopy. These 3 selected communities with near-elimination were all previously hyperendemic based on results from baseline data in 1996 (with weighted microfilaridermia prevalence values of >80%). The follow-up weighted microfilaridermia values in 2015 in these communities was 10% for Se Dembom and 0% for Lemgo and Tsesse [13]. Annual mass treatment with ivermectin commenced in this health district in 1996. Initial treatment coverage in the region as a whole was low at the beginning (30–35%) but it increased to 60–65% upon the introduction of the community directed treatment with ivermectin (CDTi) approach in 1999 [44]. Subjects were selected for the serum collection based on negative skin-snip results. Serum samples for this group were collected in June 2017.

The group with active loiasis (LLS—Loa loa serum) consisted of individuals from the loiasis endemic regions of the Mvila Division in the rain forest of the Southern Region of Cameroon in areas with high loiasis endemicity but <20% prevalence of onchocerciasis and thus without CDTI programs ongoing. This group consisted of individuals tested positive with *Loa loa* microfilariaemia of greater than or equal to 30,000 mf/ml not taking part in mass drug administration programs. The subjects in this group were recruited previously for a study on the effects of albendazole of *Loa loa* microfilaraemia as described by Kamgno *et al*., [45]. Serum samples from these *Loa loa* infected subjects were obtained from the Centre for research on filariasis and other tropical diseases, (CRFilMT), Yaoundé, Cameroon.

The onchocerciasis non-endemic group (ECS—European control serum) consisted of selected healthy Europeans aged between 20–26 years who have never been to an onchocerciasis-endemic setting and therefore have not been exposed to the parasite. These samples were collected in June 2016.

Blood samples collected from each of the subjects were processed for serum collection using a standard protocol [46], diluted 1:2 in glycerol and stored at -20°C.

In summary, for this work the serum samples used in the various evaluations were divided into six different categories:

OVS: 58 individuals who were tested mf (+) regardless of IVM intake, using the skin-snip test. Some were mf (+) were without detectable nodules while others were mf (+) and nodule (+);
ENS: 50 non-symptomatic persons with no previous ivermectin treatment record, without patent disease and skin snip negative;
HES: 50 apparently healthy persons without any onchocerciasis-related symptoms. The members of this group, born and living in Rwanda, were not parasitologically tested for *Onchocerca volvulus* infection. They had never taken IVM in their life as this disease is not a public health concern in this country.
ITS: 68 skin snip-negative persons from an onchocerciasis near-elimination (previously hyperendemic) setting. These persons were selected from 3 onchocerciasis-near elimination communities (two with microfilaridermia prevalence values of 0.0% and the 3rd with a prevalence of 10.0%). These persons were selected based on their microfilaria-negative status irrespective of ivermectin treatment status.
LLS: 50 persons actively infected with *Loa loa* based on microfilariae data; the persons in this group were not parasitologically tested for onchocerciasis.
ECS: 3 persons from onchocerciasis non-endemic settings;

Apart from the serum samples from the *Loa loa* infected subjects, all the other samples used for this work were collected purposely for this study.

### Ethical considerations and participant enlistment

The study was approved by the Cameroon National Ethics Committee for Research in Human Health, CNERSH (N°2015/01/543/CE/CNRESH/SP). Administrative authorisation was obtained from the Cameroon Ministry of Public Health (N°631–1315). Written informed consent forms were distributed and well explained to all participants who took part in the study. All the study subjects voluntarily signed and returned the informed consent form before participation in the study. For participants who were children, consent was obtained from their parents or guardians. Demographic data were recorded for each participant on questionnaire forms. Confidentiality was observed during data collection, analysis, and reporting. All study participants comprised individuals of both sexes aged 5 years and above.

Medical care was offered for diagnosed illnesses (e.g. intestinal infections; general complaints, etc) after consultation with the medical doctor.

### *Onchocerca spp* parasite material

Nodulectomy was carried out by trained medical personnel under aseptic conditions after application of local anaesthesia. Crushing of the nodules with surgical instruments was meticulously avoided and oral paracetamol was offered as postoperative analgesic. The extirpated nodules were kept in gentamycin-supplemented RPMI-1640 medium and transported to the lab where adult worms were obtained according to the method previously described [47]. Briefly, average nodular masses were digested in culture medium supplemented with 0.5 mg/mL collagenase for 9 hours at 37°C shaking at 90 rpm after which the male and female worms were cultured in incomplete RPMI-1640 supplemented with 0.25 mg/ml gentamycin and 2 mM L-glutamine.

Parasitological examination for *O*. *volvulus* microfilariae was done by a two-site skin snip from both knees and microscopic examination for the presence of mf following 12 hours of incubation. Microfilaria presence in any of the skin snips confirmed the diagnosis. The motility was checked and the numbers for the individual microfilarial densities was recorded as the arithmetic means for the two snips (mf/ss). Emerged microfilariae were pipetted and washed thrice with 20% percoll. Both microfilariae and adult worms obtained from the infected individuals were thoroughly washed and stored at -80°C for nucleic acid and protein extraction.

L2 and L3 larval stages were obtained from infected and/or infective blackflies as described elsewhere [48]. Briefly, infected *Simulium* flies were captured and grown in the laboratory for seven days after which the thorax and head regions were dissected with the aid of a dissecting microscope to obtain L2s and L3s respectively. The isolated developmental stages of the larvae (L2 and L3) not used immediately were stored separately.

### Sequence and phylogenetic analyses

Ov-47 (UniProt ID: O46146) was identified and isolated from a lambda gt-11 cDNA expression library derived from adult female *Onchocerca volvulus* mRNA using rabbit anti-serum raised against the cuticular surface proteins of *O*. *volvulus* female worms and expressed as a 359-amino acid, 47 kDa protein [27]. Ov-47 and OVOC5284 (the predicted parent protein of Ov-47) (WormBase ID) were analysed with MEMSATSVM [49,50] for the prediction of transmembrane helix domains and SignalP 4.1 [51] for the presence of a classical secretory peptide sequence. To check the predictions from the previous analyses, the proteins were further analysed with TOPCONS [52] which simultaneously detects both the transmembrane domain and signal sequence regions. Pictorial presentation of transmembrane domains and other features in proteins was done using Protter [53] with the data obtained from the preceding analyses. Protein orthologue sequences from related nematodes were retrieved from UniProt [54] and WormBase [55]. Sequence analysis for physico-chemical properties was done using Protparam [56], and the multiple sequence alignments of protein orthologs from selected nematode species was done using Multalin [57]. The phylogenetic tree of Ov58GPCR and related parasite sequences was constructed using MEGA X [58] after multiple sequence alignment with MUSCLE [59]. The evolutionary history was inferred by using the Maximum Likelihood method based on the JTT matrix-based model [60] and phylogeny was tested using the Bootstrap method. The number of Bootstrap replications was set at 500. The tree was drawn to scale, with branch lengths measured in the number of substitutions per site. All positions containing gaps and missing data were eliminated.

### Immuno-informatics analyses and peptide synthesis

Linear (continuous) B-epitopes were predicted using BCPreds [61], ABCPreds [62], SVMTrip [63] and BepiPred-2.0 [64] separately. Three of the most recurrent linear B-epitopes (designated, P1, P2 and P3) were chosen for synthesis. Initially, P1 and P2 were synthesized by GenicBio Ltd, Shangai, China and produced with at least 95% purity.

**Fig 1:**
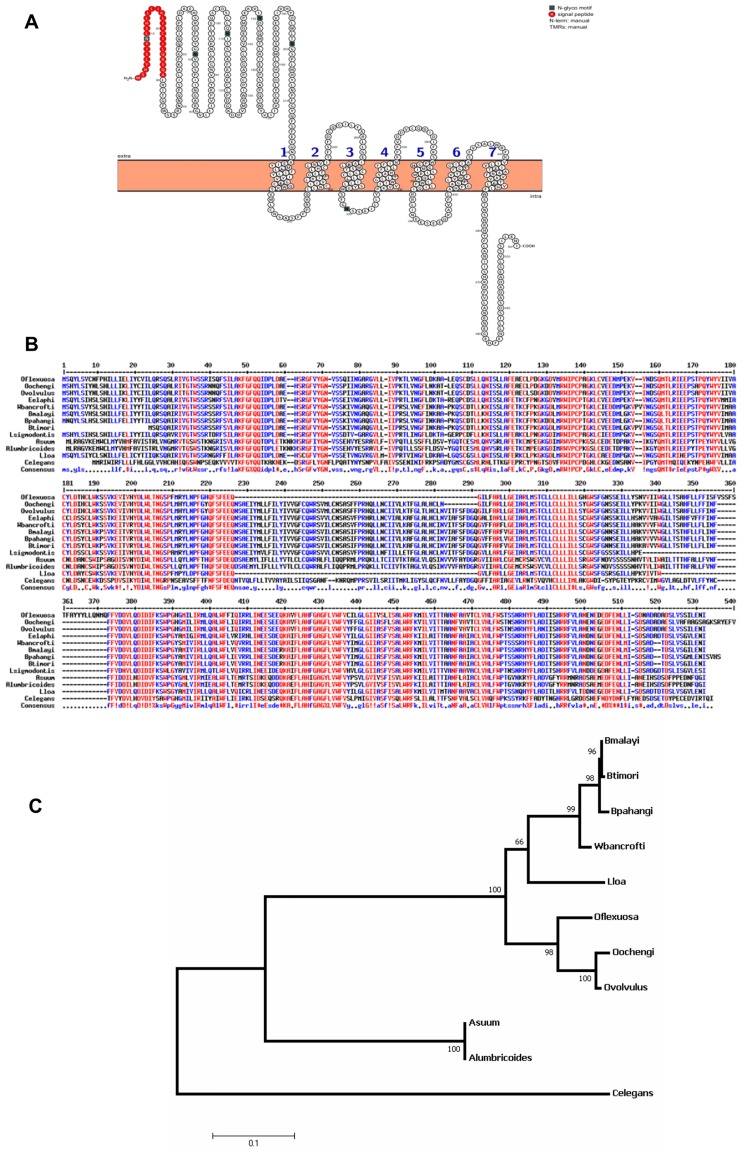
Ov58GPCR is a 7-transmembrane protein conserved in related nematodes. (A) Pictorial presentation of Ov58GPCR transmembrane topology showing the long extracellular domain and the 7 transmembrane helix regions (numbered 1 to 7) as generated by MEMSATSVM and TOPCONS and illustrated by PROTTER (Red residues indicate the signal peptide). (B) Multiple sequence alignment of Ov58GPCR with homologues in some key nematodes revealing the respective identity percentages: *O*. *volvulus (OVOC5284)*, *O*. *flexuosa (A0A183HRS3)*, **76.0%;** *O*. *ochengi (A0A182E0H8)*, **97.2**%; *Wuchereria bancrofti (A0A1I8EAM4)*, **82.3%;** *Brugia malayi (A0A0K0JJD3)* **81.5%;** *Brugia pahangi (A0A0N4TKQ9)* **81.5%;** *Brugia timori (A0A0R3QZE7)* **81.3%;**, *Ascaris suum (U1MP61)* **53.8%;** *A*. *lumbicoides (A0A0M3HY48)* **53.8%;** *Loa loa (A0A1S0TX81)* **63.5%** and *Caenorhabditis elegans (Q22162)* **44.2%;** data were retrieved from WormBase and aligned using Multalin. Consensus levels were set at the default parameters of the program: red color indicates a high consensus (90%), blue color a low consensus (50%) and black color stands for neutral. (C) The above related orthologues were used to depict the phylogenetic relationship between Ov58GPCR in *O*. *volvulus* and its homologues in other parasites. The evolutionary history was inferred by using the Maximum Likelihood method based on the JTT matrix-based model to estimate the distances. and phylogeny was tested using the Bootstrap method. The tree was drawn to scale and the Bootstrap values are indicated on the tree.

In a bid to raise antibodies specific to epitopes on the extracellular domain, the protein was further analysed by the OptimumAntigen^™^ design tool (GenScript, Piscataway, USA) based on a combination of factors such as host homology, hydrophilicity, solvent accessibility, secondary structure, flexibility, and antigenicity and on ease of synthesis. The best peptide (most immunogenic), which was a shorter version of a previously predicted and synthesized peptide P1, designated P4, was selected and synthesized with P3 initially predicted with >93% purity. Physicochemical properties of the peptides were predicted using Protparam [56] while secondary structure prediction was done using Psipred v3.3 [65] and Jpred3 [66]. The conservation percentage (for amino acid with high consensus values) of all the synthesized peptides amongst the related nematodes selected was obtained following multiple sequence alignment on Multalin (Fig 1B). P4, (CVEEDMPEKVIN) was used to immunize rabbits and following a secondary boost, immune serum was collected from the rabbits and the corresponding antibodies were further affinity-purified from the immune serum (GenScript, Piscataway, USA).

### Serological assays for peptides and peptide cocktails

IgG responses to individual synthetic peptides and various cocktail combinations were analyzed by indirect ELISA with infected individuals’ and control sera. MaxiSorp^™^ flat bottom 96-well microtiter plates (Nunc, Roskilde, Denmark) were coated with 250 ng of peptide in coating buffer (100 mM carbonate buffer, pH 9.6) and incubated at 4°C overnight. Plates were washed 3 times with PBS-0.5% Tween 20 (PBST) after each incubation step. Peptide-coated plates were blocked by incubation with 150 μL blocking agent (1% BSA in PBST) at 37°C for 1 hour 30 minutes and incubated with 50 μL human sera for diluted 1:800 in PBST at 37°C for 1 hour 15 minutes. Antibody binding was detected by incubation with 50 μL of goat anti-human IgG antibody, Fc-specific, HRP conjugate (MerckMillipore, Massachusetts, United States) diluted 1:5000 in blocking buffer at 37°C for 1 hour. The chromogen development was mediated by the addition of 50 μL substrate solution (Supersensitive 3, 3', 5, 5'-tetramethylbenzidine, SIGMA-ALDRICH). The reaction was stopped by with 50 μL of 2N sulfuric acid following 10 minutes of incubation at 37°C and the OD was read at 450 nm.

### Cloning, mass expression and purification of Ov58GPCR extracellular domain of (Ov58GPCR-ECD)

Given the nature of Ov58GPCR (a multi-pass transmembrane protein) and the difficulties associated with GPCR expression [67], the gene portion coding for the extracellular domain (ECD) without the putative signal sequence (30–222) was cloned into pET30a+ (Novagen, Gibbstown, NJ) in-frame for the expression of a C-terminal 6xHis tagged protein and the construct was used to transform SHuffle T7 Express *E*.*coli* cells (which have enhanced capacity to correctly fold proteins with multiple disulphide bonds in the cytoplasm) (New England Biolabs, Ipswich, USA). Expression was induced using 1mM Isopropyl β-D-1-thiogalactopyranoside (IPTG) at room temperature overnight, shaking at 190 rpm and the protein was expressed as a 6x-His fusion protein. The bacteria cells expressing protein were pelleted from culture medium and lysed by sonication (40% Amplitude, 10 minutes, 4°C). Due to difficulties in obtaining a soluble protein, the expressed antigen was purified from inclusion bodies. Following sonication, the bacteria pellet was solubilized using urea as described [68]. Briefly, following several washes with 50 mM Tris containing 500mM NaCl, 2% cholic acid and 2M urea, solubilisation was achieved by incubating bacteria pellet in buffer containing 2M urea at pH 12.5 overnight at room temperature. Solubilised protein was purified using Ni^++^-immobilized-metal affinity chromatographic (IMAC) columns (GE Healthcare) following standard protocols. Due to the presence of contaminating proteins, a further round of purification for pooled Ni^++^-IMAC purified fractions was achieved by size-exclusion chromatography using the HiLoad 16/600 Superdex 200 pg column on an AktaPurifier (GE Healthcare Life Sciences) after the calibrating column using protein standards containing Thyroglobulin, MW 670 kDa; Ovalbumin, MW 45 kDa; gamma-globulin, MW 93 kDa and Vit B12, MW 1.35kDa. The purified protein was resolved on SDS-PAGE and its identity confirmed by Western blot using monoclonal anti-6xHis antibodies (Sigma-Aldrich). The concentrations of purified protein fractions were determined using the BCA protein assay kit (Pierce, Rockford, IL).

For Western blots, 2μg of protein samples were run on a 12% Tris-glycine polyacrylamide gel (Bio-Rad, Carlsbad, CA) and subsequently transferred to Hybond-C Extra nitrocellulose membranes (GE Healthcare). Blocking was done either with Odyssey Blocking Buffer (PBS) (LI-COR, Lincoln, NE) or 5% milk in TBS-NP40 overnight at 4°C followed by incubation with mouse monoclonal anti-6xHis antibodies (1:4000) and anti-Ov58GPCR-ECD antibodies (1:15000) for 1 hour 30 minutes. Following three changes of wash buffer (TBS + 0.005% NP40) at 5-minute’ intervals each, membranes were incubated with the appropriate ALP-conjugated secondary antibodies (1:5000) for 1 hour 30 minutes and detected with nitro blue tetrazolium/5-bromo-4-chloro-3-indolyl phosphate (NBT/BCIP). All antibody incubation steps were done at room temperature.

### Serological characterization of Ov58GPCR-ECD

#### Total IgG and IgG subclass ELISA

To evaluate the potential role of Ov58GPCR-ECD in diagnosis and in the assessment of drug treatment programs success, individual serum samples from the 6 subject groups consisting of infected individuals and different controls collected from various populations with defined disease status—namely: OVS ((human onchocerciasis sera) for individuals actively suffering from the disease, ENS (endemic non-symptomatic sera) for people who have been living in endemic area for extended periods without developing the disease, ITS (Ivermectin treatment sera) for samples with at least 3 years of IVM treatment from the Bandjoun HD earlier mentioned, LLS (*human loiasis sera*) for individuals infected with *Loa loa* parasite, HES (African hypo-endemic sera) for a population of Rwanda where onchocerciasis is hypo-endemic, and ECS (European control sera) for disease-free people of European origin) were screened for contents of antigen-specific antibodies. The numbers of samples used for this assay were as follows: OVS (58); ENS (50); ITS (68); LLS (50); HES (50), and ECS (3). Optimal antigen/antibody concentrations were determined by checkerboard titration. MaxiSorp^™^ 96-well microtiter plates (Nunc, Roskilde, Denmark) were coated with 4 μg/mL (200 ng) of purified antigen overnight at 4°C in 100 mM carbonate buffer, pH 9.6. Plates were washed thrice with wash buffer (PBS + 0.05% Tween 20), with 5 minutes between each wash and blocked with 10 mg/mL of BSA for 1 hour 30 minutes at 37°C. The plates were washed as previously described and the various serum samples were incubated as primary antibody at a dilution of 1:1000 for 1 hour at 37°C. Goat anti-human IgG (Fc specific) peroxidase conjugate (Merck Millipore, Billerica, MA) was incubated as secondary antibody at a dilution of 1:5000 for 1 hour at 37°C. After a final wash, the chromogenic substrate, TMB was incubated as the substrate for 10 minutes at 37°C. The reactions were stopped with 2N H_2_SO_4_ after which optical densities were recorded at 450 nm using the SPECTROstar Nano (BMG LABTECH, Ortenberg, Germany). All washings and antibody dilutions were done in wash buffer (PBS+0.05%Tween-20). IgG subclass responses were conducted for endemic non-symptomatic and infected subjects (since these subclass responses have been reported to provide more information on parasitological status in onchocerciasis) [69] as described above except for the use anti-human IgG subclasses as secondary antibody and an additional incubation step of goat anti-mouse IgG peroxidase conjugate as tertiary antibody. For subclass responses, monoclonal mouse anti-human IgG1, IgG2, IgG3 and IgG4 antibodies were diluted 1:2000 and the HRP-conjugated anti-mouse IgG was also diluted 1:2000. All antibodies were purchased from Sigma-Aldrich (St. Louis, MI, USA).

#### Antigen-specific IgE ELISA

Sera pools from subject groups (except for the ECS group) previously described for antigen-specific IgG response were used. Ov58GPCR-ECD specific IgE responses for these different groups of subjects were evaluated by slightly modifying the method previously described [70]. The samples were initially treated with anti-human IgG (to reduce the IgG concentration in the sample and mitigate the IgG/IgE antibody competition). The sera pools (each consisted 5 individual serum samples) were diluted with an equal amount of the IgG-absorbent, mixed and incubated for 1 hour at room temperature and then overnight at 16 °C. Ten pools were screened for each group while 3 individual samples for the ECS were also screened. Immune complexes were thereafter removed by centrifugation at 7000 rpm for 15 minutes. The remaining supernatants were transferred to new tubes and used for IgE-content analyses.

The standard test configuration with a 4-step procedure was used, with 3 antibody steps. Briefly, 96-well polystyrene microtiter plates (MaxiSorp^™^ 96-well microtiter plates, Nunc) were coated with the purified antigen (4 μg/mL in 0.1 M carbonate buffer, pH 9.6) and incubated overnight at 4 °C. The plates were washed with washing solution (0.05% Tween- 20 in phosphate buffer (PBS), pH 7.4, PBST and blocked with 1% BSA in PBS pH 7.4 for 1 hour 30 minutes at 37 °C. After the washing step, 50 μL per well of each 1:30 diluted serum pool was added in duplicates and incubated for 2 hours at 37 °C. After 3 washes with 5-minute intervals, 50 μL per well of mouse anti-human IgE antibodies was added at 1:2000 dilution to each well and incubated for 1 hour at 37°C. This step was followed by another wash and then incubated with anti-mouse IgG-HRP conjugate at 1:4000 for 1 hour at 37 °C. After a final wash, 50 μL per well of chromogenic solution (3,3′,5,5′-tetramethylbenzidine hydrogen peroxide–TMB substrate) was added and the plates were incubated at room temperature for 10 min. The reactions were stopped with 2N H_2_SO_4_ after which optical densities were recorded at 450 nm using the SPECTROstar Nano (BMG LABTECH, Ortenberg, Germany).

#### Antibody responses and compliance to ivermectin

To evaluate the potential role of Ov58GPCR-ECD in the monitoring progress of chemotherapeutic programs, all sera from the ITS group (Bandjoun HD) was subdivided into 4 sub-categories, according to years of treatment with ivermectin and tested for total IgG and IgG subclass responses as described. The goal of the experiment was to assess the effects of drug treatment on antigen-specific antibodies in the microfilaria-negative individuals. These 3 targeted communities were previously reported with high microfilaridermia (>80%) [13]. Following around 20 years of IVM MDA, the results of the studies carried out in 2015 [13] motivated us to select the three communities for the above objective in this study. Serum samples from individuals from all three communities were used in this assay. Individual serum samples were depleted of IgG and used to determine antigen-specific IgE response as previously described. Ten samples were used per group to measure levels of total IgG and antigen-specific IgE while the number of samples used for the measurement of IgG-subclass levels was 16 for each group.

### Stage-specific expression

Total RNA was extracted from the different *O*. *volvulus* parasite stages using the RecoverAll^™^ Total Nucleic Acid Isolation Kit (Life Technologies, Carlsbad, CA, USA). RNA quality and quantity were assessed with the NanoDrop ND-1000 spectrophotometer (NanoDrop Technologies, Wilmington, DE). The RNA was reverse transcribed to cDNA using the iScript^™^ Select cDNA Synthesis Kit (BioRad, Hercules, CA, USA). The introns and exons structure of the *Ov58GPCR* gene was illustrated using the gene structure display server, GSDS 2.0 [71]. Two pairs of primers were designed for amplification across adjacent exons. The first primer set predicted to amplify a product spanning exon 2 and exon 3 (Forward primer, F2, 5’ ATTCCGTGTCCAGCAGGAA 3’, Reverse primer, R3, 5’ CATCAGCTCCTCAATATTGG 3’) was designed to produce amplicons of 390 bp and 111 bp from genomic and cDNA, respectively. The second primer set predicted to amplify a product spanning the 8^th^ and 9^th^ exons (Forward primer, F8, 5’ GGCGGCAAATTTTGTTGCA 3’, Reverse primer, R9, 5’ CTGATGCGGAGAGCCTTGTT 3’) was designed to produce amplicons of 338 bp and 187 bp from genomic and cDNA respectively. PCR amplification was done with the T100 Thermal Cycler (BioRad^™^, Hercules, CA, USA) under the following conditions: 94°C for 2 min, 1 cycle; 94°C for 10 s, 55°C for 10 s and 72°C for 1 min, 40 cycles; 72°C for 10 min. The reaction mix was prepared using the ReadyMix^™^ REDTaq^®^ PCR reaction mix (SIGMA-ALDRICH, St. Louis, MI, USA) according to manufacturer’s protocol, The PCR products were run on a 1% agarose gel, stained with SYBR Safe (Life Technologies, Carlsbad, CA, USA) and visualized under UV light using the Gel Doc XR+ (BioRad^™^, Hercules, CA, USA).

Total protein extracts were prepared from the worms as described previously [72] with slight modifications. Worm pools from the different parasite stages were homogenized in a Porcelain mortar with a Teflon pestle in RIPA buffer (10 mM Tris-HCl, pH 7.4, 150 mM NaCl, 1% Nonidet P-40, 0.2% sodium deoxycholate, 1 mM EDTA and 10 mM NaF). The homogenate was centrifuged, and protein in the supernatant was measured using the Pierce BCA assay (Thermo Fisher Scientific, Rockford, IL).

Worm cuticular surface proteins were prepared as previously described [73] with some modifications. Briefly, worms were incubated in phosphate-buffered saline (PBS), pH 7.4 with 0.25% cetyltrimethylammonium bromide, CTAB (SIGMA, USA) and 1% sodium deoxycholate (SIGMA, USA) at 16°C overnight. The supernatant was obtained by centrifugation at 4°C, 11,000 × g for 20 min and concentrated by acetone precipitation. The concentrated protein following acetone precipitation was solubilized in 100mM Tris-HCl pH 8.5 and stored at -20°C.

Protein samples were loaded onto a 12% SDS-PAGE gels and run at 190 V. Following SDS-PAGE, the proteins were transferred to nitrocellulose membranes and blocked with Odyssey Blocking Buffer (PBS) overnight at 4°C. Incubation was done with anti-Ov58GPCR-ECD antibodies at a dilution of 1:7,500 for 1 hour 30 minutes. After three washes in TBS-NP40, the membranes were incubated with rabbit alkaline phosphatase-conjugated secondary antibody diluted at 1: 5,000 (Promega, Madison, WI) for 1 hour 30 minutes. Following three washings with wash buffer, the membranes were detected either with NBT/BCIP. All antibody steps were done at room temperature.

### Data analyses

Normality of distributions was assessed using a Shapiro-Wilk test. Comparisons of more than two groups were made using a one-way ANOVA (with Holm-Sidak’s procedure for multiple comparisons) or a Kruskal-Wallis test (with Dunn’s or Tukey’s correction for multiple comparisons) for independent groups as appropriate. 95% confidence intervals on the difference between means were also computed. Comparisons of more than two groups in case of paired data were made using repeated measures ANOVA on ranks. The diagnostic performance of the total IgG was assessed for each study peptide using receiver operating curve (ROC) analyses. Thirty-two samples each were used for the groups analyzed. Areas under the ROC curves (AUCs) were evaluated using the trapezoid method. Standard errors of the AUCs were calculated using the procedure of DeLong *et al* [74]. Exact 95% confidence intervals for the AUCs were determined using a binomial approach. For each peptide, an optimal cutoff value was selected according to the highest Youden’s index for that peptide. Sensitivities, specificities, positive and negative likelihood ratios (with 95% confidence intervals) were then calculated for the selected cutoff values. A p value < 0.05 was considered statistically significant. All calculations were performed using SigmaPlot^®^ for Windows, version 12.5 (Systat Software Inc., Chicago, IL, USA) and MedCalc^®^ Statistical Software version 18 (MedCalc Software bvba, Ostend, Belgium)

## Results

### Ov58 is a conserved GPCR in nematodes

Ghogomu *et al*., [27] previously reported the characterisation of a protein identified using a lambda gt-11 cDNA expression library derived from adult female *Onchocerca volvulus* mRNA screened with rabbit antiserum raised against the cuticular surface proteins of *O*. *volvulus* female worms. This protein was named Ov-47 by virtue of its apparent molecular weight as revealed by SDS-PAGE. Advancement in functional genomics and genome sequencing of several nematode species motivated further analyses of this antigen. A BLAST search in WormBase against the *O*. *volvulus* genome using the Ov-47 sequence (359 amino acids) as a query hit the protein OVOC5284 composed of 506 amino acids accounting for a theoretical molecular weight of 58 kDa. Pfam analysis of the latter detected a Rhodopsin-like GPCR transmembrane (TM) domain. Other bioinformatics investigations detected 7 TM domains as well as an extracellular N-terminal domain and an intracellular C-terminal domain typical to GPCRs. This *O*. *volvulus* protein was therefore renamed Ov58GPCR. Thus, the structural features of Ov58GPCR were illustrated using PROTTER (Fig 1A) also showing a signal peptide sequence (marked in red) at the amino-terminal end of the extracellular domain (ECD). Extended *in silico* investigation unveiled that Ov58GPCR comprises 14 Cysteine residues, 8 of which occupy similar positions in its homologues in related nematodes such as *Onchocerca ochengi*, *Brugia timori*, *Loa loa*, *Brugia pahangi*, *Brugia malayi*, *Wuchereria bancrofti*, and *Caenorhabditis elegans* (Fig 1B). The degree of relationship of Ov58GPCR with its homologues in related nematodes is illustrated in the phylogenetic tree (Fig 1C).

### The extracellular domain (ECD) of Ov58GPCR harbours antigenic features

**Fig 2:**
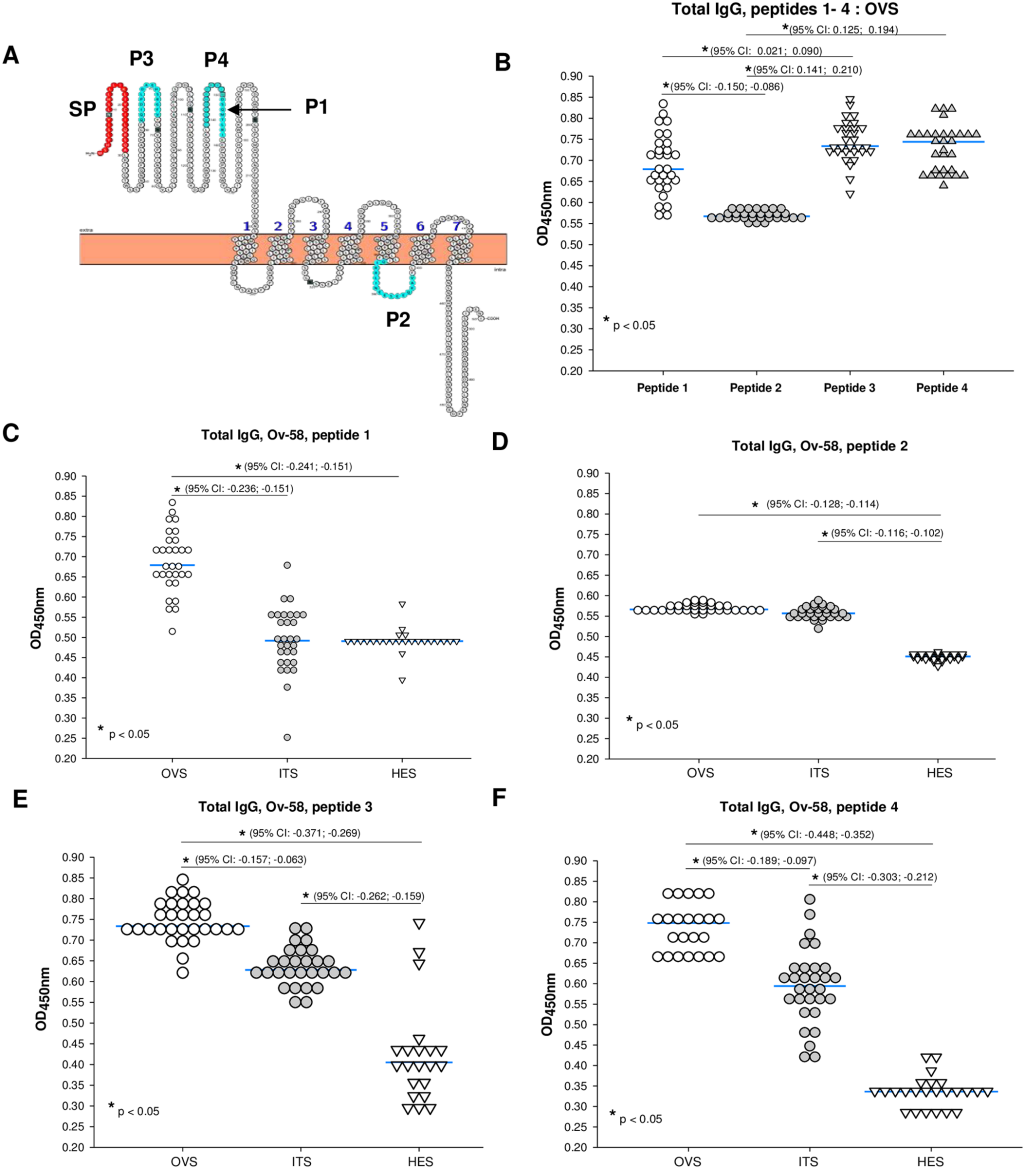
Ov58GPCR harbours immunogenic epitopes in its extracellular domain. (A) Serum samples collected from all participants (OVS, ITS and HES) were investigated for the presence of epitope-specific IgG against 4 linear B-epitopes of Ov58GPCR (P1, P2, P3 and P4 (a shorter version of P1) predicted using bioinformatics tools (see Table 1) and selected for synthesis. (B) ELISA plates were coated overnight with the indicated synthetic peptide. IgG responses to each peptide were assessed for all individual samples diluted 1:800 from infected persons (OVS) initially to evaluate the immunoreactivity of each epitope. (C-F) Thereafter, the ability of each of the peptides to distinguish between infected individuals and controls was tested using individual serum samples from *Onchocerca volvulus*-infected individuals (OVS) and controls (ITS & HES). Following ANOVA analysis, p-value less than 0.05 was considered statistically significant. OVS: *Onchocerca volvulus serum* or Human onchocerciasis serum (serum from actively infected people), ITS: Ivermectin Treatment Serum (serum from an onchocerciasis near-elimination setting), HES: serum from an onchocerciasis-hypoendemic setting). The confidence intervals are indicated.

In order to investigate the immunogenicity of Ov58GPCR, the protein sequence was subjected to *in silico* analysis using different online epitope prediction servers. It was hypothesized that extracellular epitopes (from the extracellular domain and extracellular loops) elicit stronger immune responses in infected individuals since they are more exposed to the immune system compared to those located the in cytosolic region. The peptides, P1 and P2 (Fig 2A) were first selected, synthesized (GenicBio Ltd, Shanghai, China) and analysed by ELISA. Results obtained confirmed the hypothesis as P1 elicited significantly higher total IgG responses compared to P2 (Fig 2B). To further assess this hypothesis, peptides P3 and P4 (a shorter version of the previously predicted and synthesized peptide P1) were purchased (GenScript, Piscataway, NJ, USA) and analysed in a similar manner. The physicochemical properties of the peptides as well as their degrees of conservation in the related nematodes used in constructing the phylogenetic tree are shown in Table 1. Total IgG responses similar to those elicited against P1 were recorded for P3 and P4 (Fig 2B), suggesting that Ov58GPCR harbours immunogenic features. In order to assign the specificity of the elicited IgG responses, serum samples from individuals with active infection, as defined by the positive skin snips (OVS), and sera from persons treated continuously for three to thirteen years or more with ivermectin (ITS) were analysed by ELISA for total IgG responses. Here, sera from a Rwandan population where onchocerciasis is hypo-endemic (HES) were used as negative controls. Results obtained revealed a significant difference in IgG levels between infected individuals and healthy African controls. As opposed to P2, the other peptides (P1, P3 and P4) all discriminated significantly between the infected population and control populations (Figs 2C–2F). For subsequent downstream applications, P4, which structurally overlapped with P1 and showed the highest reactivity was chosen to generate polyclonal antibodies (Fig 2B).

**Table 1:** Physicochemical properties of predicted B-epitopes.

Peptide	Mw (Da)	Secondary structure type	% conservation	pI	AA^b^	GV	AI
P1: CVEEDMPEKVINDSQMTLRI	2350.70	Coil/strand	45.0	4.18	139–158	-0.425	87.5
P2: IRRLINEESEEQKAV	1814.03	Helix	33.3	4.95	383–398	-1.060	104.0
P3: QQIDPLDAEHSRGFV	1711.85	Coil	40.0	4.54	52–66	-0.727	78.0
P4: CVEEDMPEKVIN	1405.60	Coil	33.3	4.0	139–150	-0.475	80.8

For linear epitopes, peptides 1, 2 and 3 were predicted using 4 tools selected based on different methods of epitope prediction and the 3 most recurrent were selected and synthesized. Peptide 4 was obtained by prediction with OptimumAntigen^™^ from GenScript. Physicochemical properties for the peptides was predicted using Protparam while secondary structure prediction was done using Psipred v3.3 and Jpred3. (Mw = molecular weight, pI = isoelectric point, AA^b^ = amino acid stretch, GV = Grand average of hydropathicity, AI = Aliphatic index. These four linear epitopes (A) were synthesized for immunological assays.

### Predicted synthetic peptide cocktails exhibit potential for use in assessing success of CDTI programs

**Fig 3:**
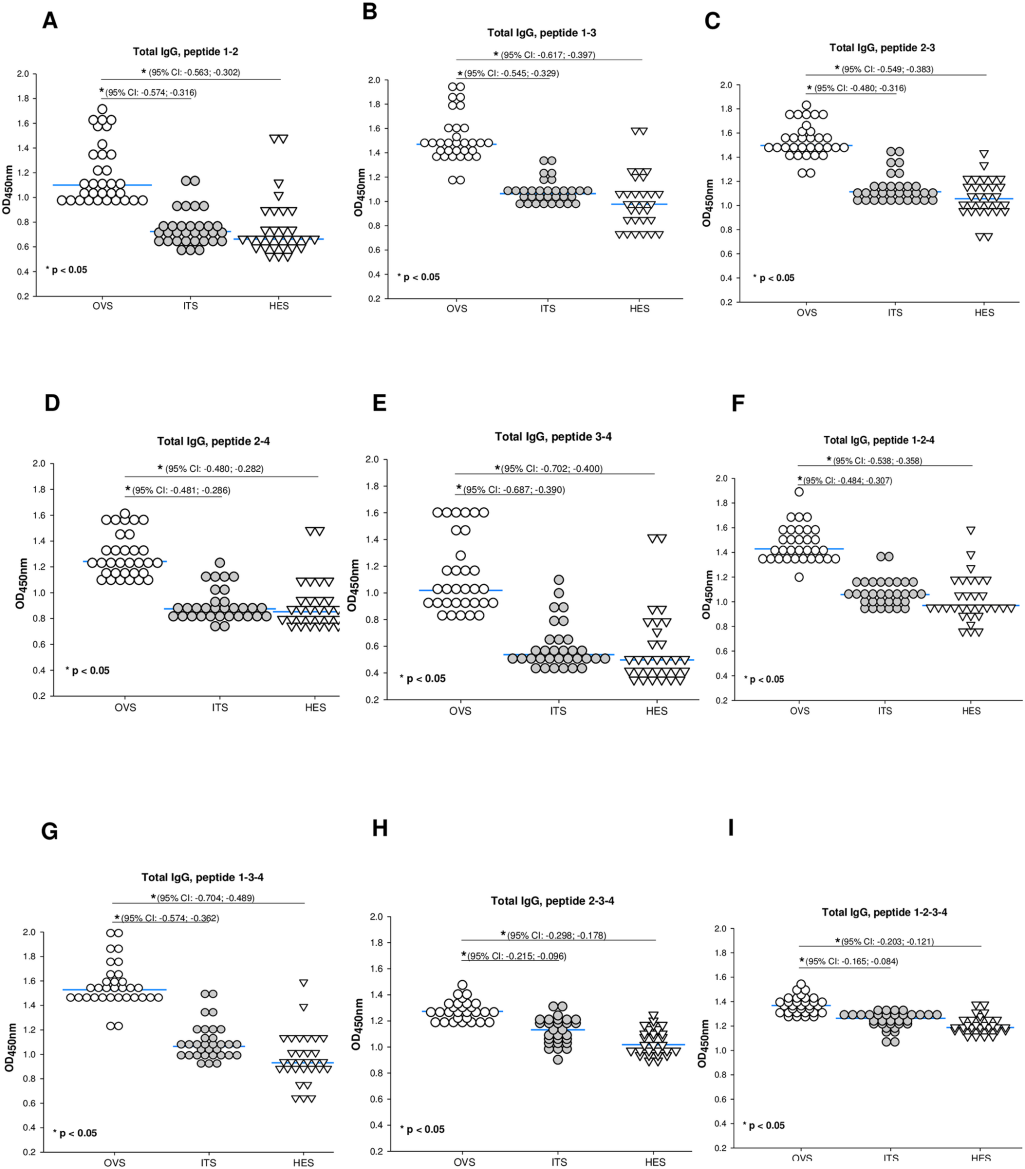
Increased antigen-specific antibody responses to Ov58GPCR peptide cocktails are associated with onchocerciasis infection. ELISA plates were coated with the different indicated peptide combinations (A-I). The combination P1-P4 was not considered as the two sequences overlap. IgG responses to the peptide cocktails were measured for all individual serum samples diluted 1:800 for infected persons and the controls. OVS: Human onchocerciasis serum, ITS (Ivermectin treatment serum, HES:hypo-endemic African serum). Following ANOVA analysis, p-values less than 0.05 were considered statistically significant. The confidence intervals are indicated.

Community-directed treatment with ivermectin (CDTI) programs have been going on for two decades in some communities. Measures to certify disease elimination and perform surveillance are still needed in health systems of countries where onchocerciasis is a public health problem. From the results above showing that some peptides of interest discriminated actively infected (OVS) and treated (ITS) persons, different peptides were combined into cocktails and their efficiency evaluated based on previous reports [75,76]. Sera samples from healthy Africans (HES—from a hypo-endemic setting) were used as controls. To this end, peptides were combined as indicated in Fig 3 and their responses to total IgG investigated by ELISA. In all the combinations, sera from *O*. *volvulus* infected individuals were used as positive controls. Results obtained indicated that almost all the cocktails amplified the signals with increased reactivity (Fig 3). The average OD450nm increased from around 0.7 for single peptides (P1, P3 and P4; Fig 2B) to almost the double with OVS-IgG responses, suggesting an increased reactivity which could be indicative of a higher sensitivity upon combining the peptides. Strikingly, while significant differences were observed between ITS and HES with individual P2, P3 and P4 peptides (Fig 2D–2F), the cocktails elicited, except for P2-3-4 (Fig 3H), similar total IgG responses in treated persons (ITS) and healthy African controls (HES). The diagnostic parameters of the different peptides and peptide cocktails are reported in Table 2.

**Table 2:** Diagnostic accuracy parameters for Ov58GPCR peptides and peptide cocktails.

Peptide	AUC	SE	95% CI for ROC AUC	p value	Selected cutoff	Sensitivity for selected cutoff and 95% CI		Specificity for selected cutoff and 95% CI		LR+ and 95% CI for selected cutoff		LR- and 95% CI for selected cutoff	
**Peptide 1**	0.977	0.0135	[0.918–0.997]	<0.0001	0.5599	96.67	[82.8–99.9]	92.59	[82.1–97.9]	13.05	[5.1–33.6]	0.036	[0.005–0.2]
**Peptide 2**	0.858	0.0399	[0.766–0.924]	<0.0001	0.5581	93.75	[79.2–99.2]	77.78	[64.4–88.0]	4.22	[2.5–7.0]	0.08	[0.02–0.3]
**Peptide 3**	0.952	0.0222	[0.880–0.988]	<0.0001	0.687	93.33	[77.9–99.2]	89.8	[77.8–96.6]	9.15	[4.0–21.1]	0.074	[0.02–0.3]
**Peptide 4**	0.951	0.0241	[0.876–0.987]	<0.0001	0.6535	100	[85.2–100.0]	90.74	[79.7–96.9]	10.8	[4.7–24.9]	0	-
**Peptide 1–2**	0.939	0.0256	[0.870–0.978]	<0.0001	0.946	100	[89.1–100.0]	90.32	[80.1–96.4]	10.33	[4.8–22.1]	0	-
**Peptide 1–3**	0.967	0.0181	[0.908–0.993]	<0.0001	1.3347	93.75	[79.2–99.2]	96.77	[88.8–99.6]	29.06	[7.4–114.0]	0.065	[0.02–0.2]
**Peptide 2–3**	0.987	0.0074	[0.939–0.999]	<0.0001	1.2686	93.75	[79.2–99.2]	90.32	[80.1–96.4]	9.69	[4.5–20.8]	0.069	[0.02–0.3]
**Peptide 2–4**	0.951	0.0217	[0.886–0.985]	<0.0001	1.095	96.87	[83.8–99.9]	88.71	[78.1–95.3]	8.58	[4.3–17.3]	0.035	[0.005–0.2]
**Peptide 3–4**	0.95	0.0218	[0.884–0.984]	<0.0001	0.7893	100	[89.1–100.0]	87.1	[76.1–94.3]	7.75	[4.1–14.8]	0	-
**Peptide 1-2-4**	0.97	0.0166	[0.912–0.994]	<0.0001	1.1941	100	[89.1–100.0]	91.94	[82.2–97.3]	12.4	[5.4–28.7]	0	-
**Peptide 1-3-4**	0.972	0.0155	[0.915–0.995]	<0.0001	1.2237	100	[89.1–100.0]	90.32	[80.1–96.4]	10.33	[4.8–22.1]	0	-
**Peptide 2-3-4**	0.93	0.026	[0.853–0.974]	<0.0001	1.1743	100	[87.7–100.0]	76.79	[63.6–87.0]	4.31	[2.7–6.9]	0	-
**Peptide 1-2-3-4**	0.929	0.0246	[0.858–0.972]	<0.0001	1.3012	84.37	[67.2–94.7]	87.5	[76.8–94.4]	6.75	[3.5–13.1]	0.18	[0.08–0.4]

Parameters were determined based on previous description [77]. Cut off values for the sensitivity/specificity pairs were obtained using a receiver operator characteristics (ROC) curve analysis of sensitivity (y-axis) against 1-specificity (x-axis) using the OVS group as patients and the ITS group as controls. Thirty-two samples each were used for the groups analyzed. ROC curves were drawn using MedCalc^®^ Statistical Software version 18. Sensitivity refers to the probability of getting a positive test result in subjects with the disease; Specificity is the probability of a negative test result in a subject without the disease. Likelihood ratio for positive test results indicates how much more likely the positive test result is to occur in subjects with the disease compared to those without the disease; Likelihood ratio for negative test results represents the ratio of the probability that a negative test result will occur in subjects with the disease to the probability that the same result will occur in subjects without the disease. The area under the ROC curve (AUC) is a measure of diagnostic accuracy. Generally, an AUC of 0.9–1.0 indicates an excellent diagnostic accuracy. SE: Standard error, AUC: Area under the ROC curve, LR+: Positive likelihood ratio, LR-: Negative likelihood ratio.

Taken altogether, it can be concluded that selected peptide cocktails could serve as molecular tools to assess treatment successes and as potential tools to certify onchocerciasis elimination in combination with the current immunological and xeno-monitoring strategies.

### Bacterially-expressed and purified Ov58GPCR-ECD revealed predominantly monomeric but also dimeric forms

**Fig 4:**
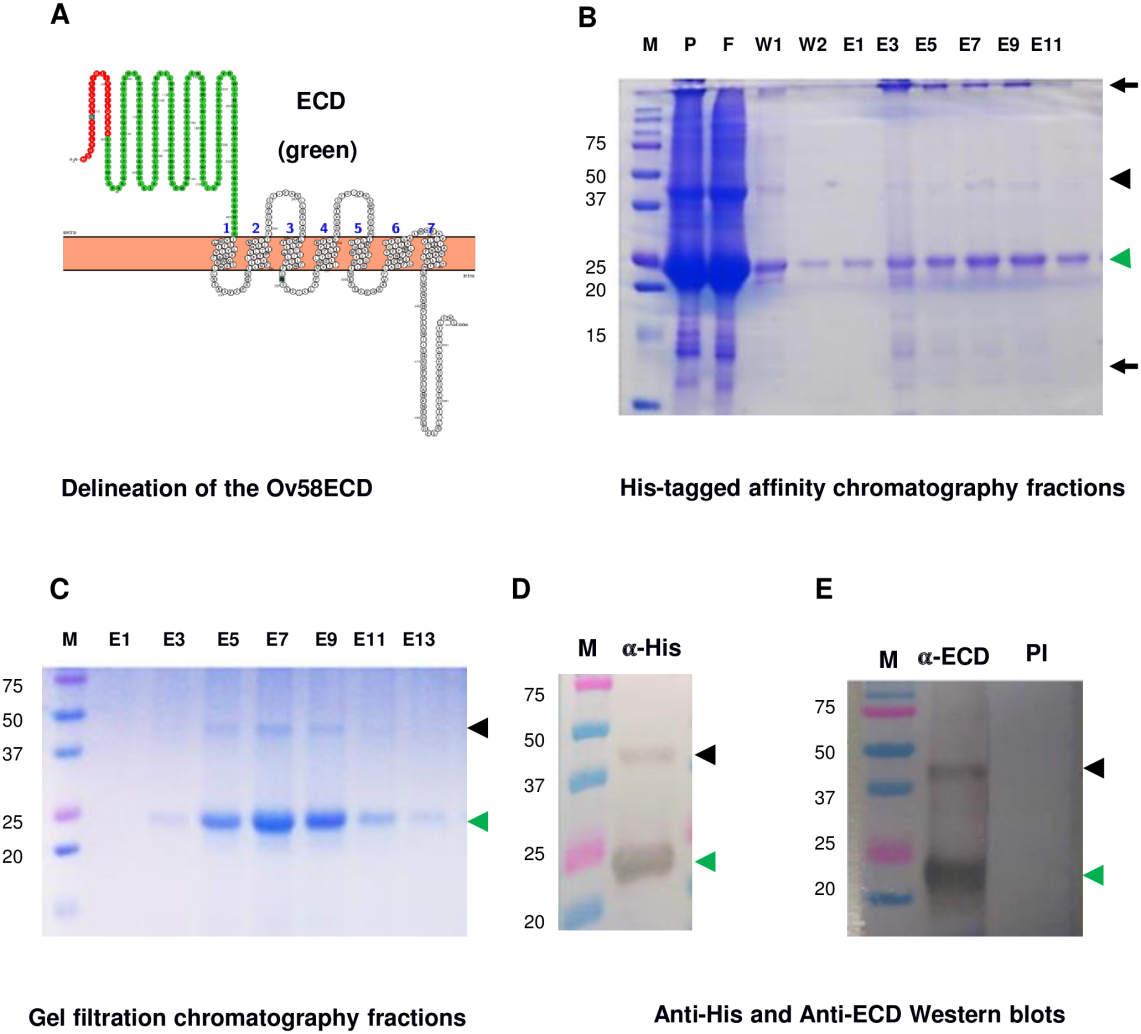
The recombinant extra-cellular domain of Ov58GPCR (Ov58GPCR-ECD) can be found in monomeric and dimeric forms. (A) The gene fragment coding for the extra-cellular domain of Ov58GPCR (green portion), without the predicted signal peptide (red portion) was cloned in pET30a+ and the construct was expressed in *E*. *coli* cells. (B) and (C) The protein, initially purified on a Ni^++^-IMAC column (B) and further purified by size exclusion chromatography (C) was resolved on a 12% SDS-PAGE gel and stained with Coomassie blue,. (D) Anti-6xHis monoclonal antibodies and (E) anti-Ov58GPCR-ECD antibodies detected two forms of the protein on nitrocellulose membranes at molecular weights of approximately 23 kDa and 46 kDa matching the expected monomeric and dimeric forms respectively. Green and black arrow heads indicate the positions of the putative monomeric and dimeric forms of Ov58GPCR-ECD respectively. Arrows indicate contaminants. M: protein ladder, P: pellet, F: flow-through, W: wash, E: Eluate, E(x): Eluate Fraction, PI: pre-immune serum, α-HIS: anti-His and α-ECD: anti-ECD.

In our ultimate goal to assess Ov58GPCR as a putative molecular diagnostic or drug target tool we focused our study on its extracellular domain (ECD). In order to use Ov58GPCR in antigen detection tests two antibodies (preferentially targeting different epitopes) are needed. Since the rabbit anti-P4 antibodies were raised initially, the generation of polyclonal antibodies in a species different from rabbit against the Ov58GPCR-ECD was desired. In this context, bioinformatics analyses were used to delineate the ECD without the signal peptide (Fig 4A; portion in green). The ECD was cloned into the pET30a+ vector and the recombinant plasmid used to transform SHuffle T7 Express *E*. *coli* bacteria, reported to be efficient for the expression of cysteine-rich proteins [78]. Expression was subsequently induced in transformed bacteria cultures, and the polypeptide of interest purified by Ni^++^immobilised-metal affinity chromatography (Fig 4B). To eliminate contaminants (black arrows), an additional gel filtration chromatography step was performed (Fig 4C). Next, the identity of the purified protein (molecular weight and purity) was assessed by SDS-PAGE and western blot using monoclonal anti-6xHis antibodies. As illustrated in Fig 4D, the western blot signals (green and black arrow heads) confirmed the two bands specifically detected on the SDS-PAGE gel (Fig 4B and 4C), which are likely to be the monomeric (green arrow heads) and dimeric forms (black arrow heads) of the purified ECD. The identities of the 2 bands detected in the purified protein fraction were further confirmed by MALDI-TOF analysis (data not shown). Finally, the ECD was used to produce polyclonal antibodies (GenScript, USA). The western blot analysis using this reagent gave a similar signal pattern to the one generated by the anti-His results (Fig 4E).

### The humoral response to Ov58GPCR-ECD divulges its diagnostic potentials

**Fig 5:**
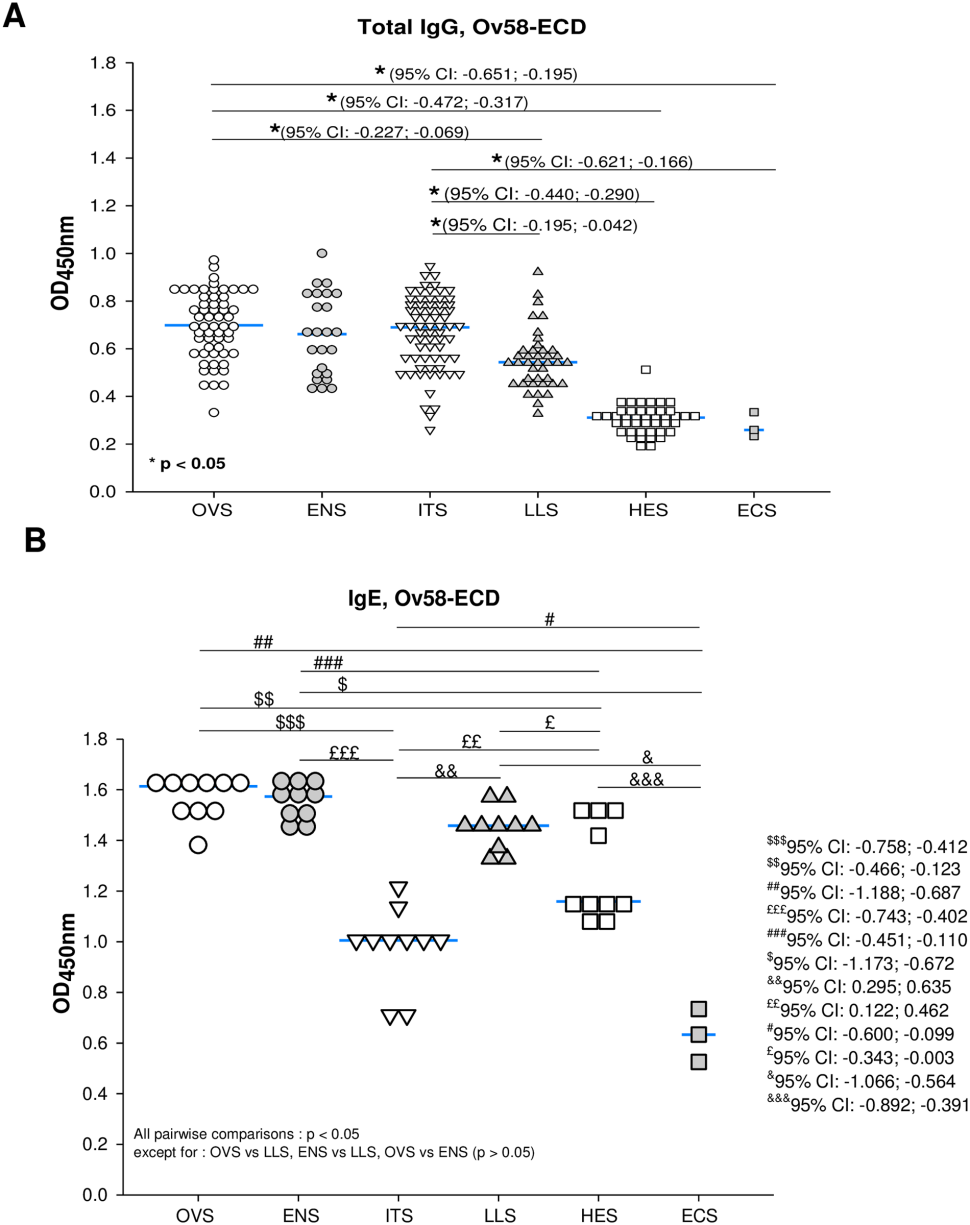
Exposure of host to parasite transmission leads to increased Ov58GPCR-ECD-specific IgG but not IgE response following treatment. ELISA plates were coated with purified recombinant protein at 4 °C. Humoral responses were measured in all individual serum samples diluted 1:1000 for (A) IgG and 1:30 for (B) IgE (after IgG depletion) in all infected persons and controls. OVS = Human Onchocerciasis Serum (58); ENS = Endemic Non symptomatic Serum (24); ITS = Ivermectin treatment serum (68); LLS = Human Loiasis Serum (50); HES = Hypo-endemic African Serum (50), and ECS = European Serum Control (3). Following ANOVA analysis, p-values less than 0.05 were considered statistically significant. The confidence intervals are indicated. The number of samples selected for each group is indicated into brackets.

We next assessed recombinant Ov58GPCR-ECD as an antigen to design a diagnostic antibody-detection test for onchocerciasis. As a prerequisite, it should display excellent specificity and sensitivity. Ov58GPCR-ECD-specific total IgG and IgE responses of the different serum groups described were investigated by ELISA. Results obtained show, as opposed to peptide cocktails reported above, that OVS, ENS and ITS groups reacted similarly with the ECD-antigen (Fig 5A).

*Loa loa* infections, because of side effects from ivermectin treatment [79], are a threat to Community-based Distribution Treatment with Ivermectin. We therefore investigated if Ov58GPCR-ECD could discriminate sera from *O*. *volvulus* and *L*. *loa* infected individuals. Sera from those actively-infected with *Loa loa* (LLS) and those infected with Onchocerca (OVS) gave significantly different responses (Fig 5A). Both the HES (sera from an onchocerciasis-hypo-endemic setting) and ECS (sera from a non-endemic setting) groups exhibited significantly lower responses compared to the other groups (Fig 5A).

Helminth infections were reported to be associated with induced eosinophilia and extensive IgE release [80] and disease clearance reverses the immunoregulatory effects, terminating the state of active suppression maintained by the parasite [81]. With that in mind, IgE responses to Ov58GPCR-ECD were addressed. Results obtained revealed no significant differences between the OVS, ENS, and LLS groups, which reacted similarly with significantly higher responses. Interestingly, a significant drop in IgE-responses was detected in sera from ivermectin treated individuals (ITS). Moreover, the HES group displayed significant recognition of the ECD (Fig 5B), suggesting possible cross-reactions. The significantly lower antigen specific IgE response recorded from the ECS group (Fig 5B) supported the specificity of the immunological reactions.

### Total IgE and IgG subclasses but not total IgG responses to Ov58GPCR-ECD negatively and significantly correlated with ivermectin treatment length

**Fig 6:**
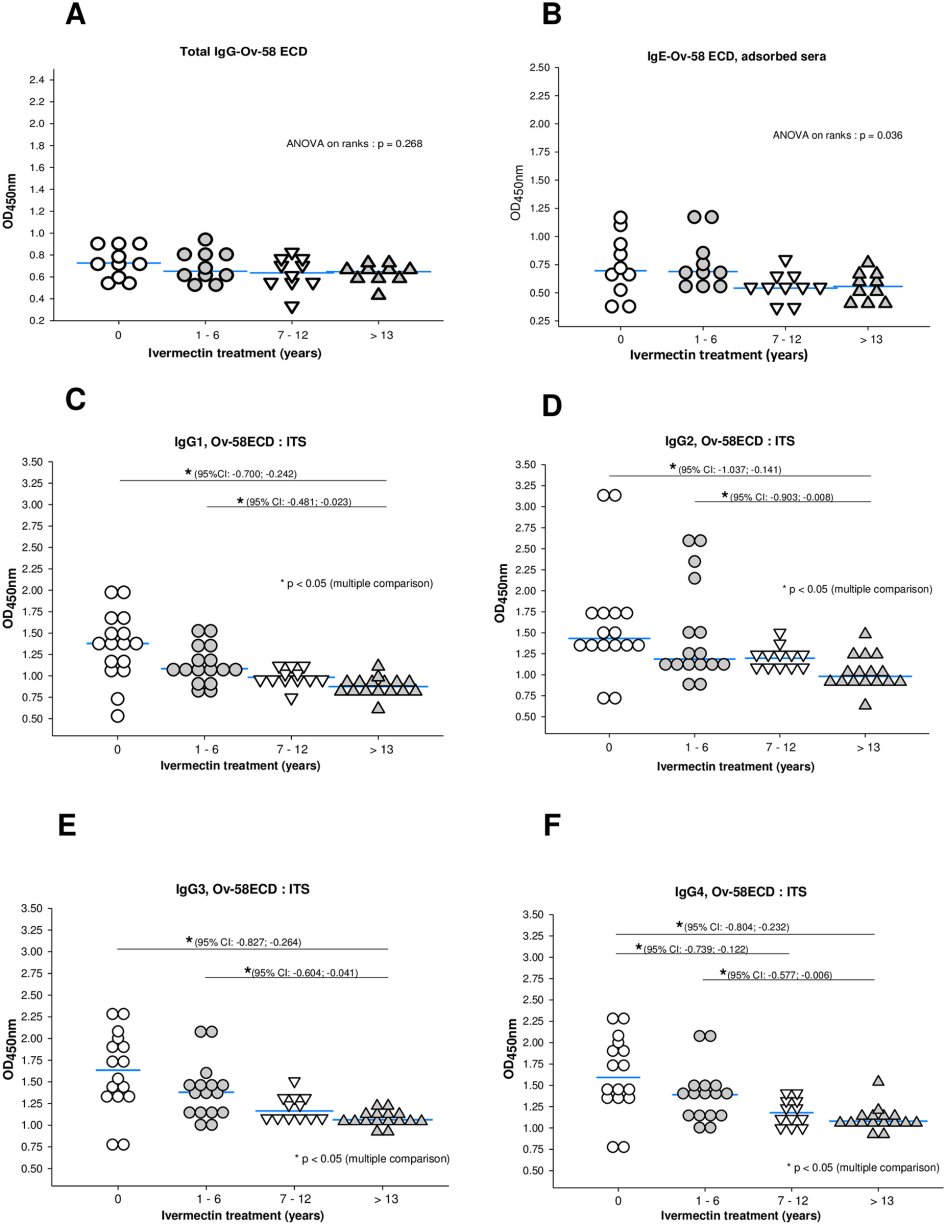
Increased ivermectin treatment length is associated with decline in IgG subclasses and total IgE responses to Ov58GPCR-ECD. ELISA plates were coated with purified recombinant protein and incubated overnight at 4°C. Total IgG responses were measured in all individual serum samples of the ITS group diluted 1:1000 for IgG (A) and 1:30 for IgE (B) and for IgG subclasses (Figs 6C-6F) after grouping sera according to years of ivermectin treatment. Following ANOVA analysis, p-values less than 0.05 were considered statistically significant. (0: never taken ivernectin, 1–6: taken ivermectin from 1 to 6 years, 7–12: taken ivermectin for between 7 and 12 years, >13: taken ivermectin for 13 years and above).

In an effort towards the design of an antibody-capture test for monitoring onchocerciasis control programs and to further investigate the observations reported above on the OVS and ITS groups (Fig 5) where similar total IgG but different IgE responses were observed, Ov58GPCR-ECD was characterized with respect to chemotherapy treatment, targeting total IgG, IgG subclasses and IgE. Samples from the ITS group were sorted as indicated (Fig 6) into different time ranges of repeated treatments. ELISA analysis confirmed previous findings obtained showing no significant fall in total IgG responses following repeated chemotherapy (Fig 6A). In contrast to total IgG, ELISA data indicated a proportionate decline in antigen-specific IgE responses with increased length of ivermectin treatment (Fig 6B). Moreover, IgG subclasses to ECD, as opposed to total IgG, also revealed similar proportionate declines with ivermectin treatment length (Fig 6C–6F).

### Ov58GPCR is expressed in larvae and adult parasitic stages

**Fig 7:**
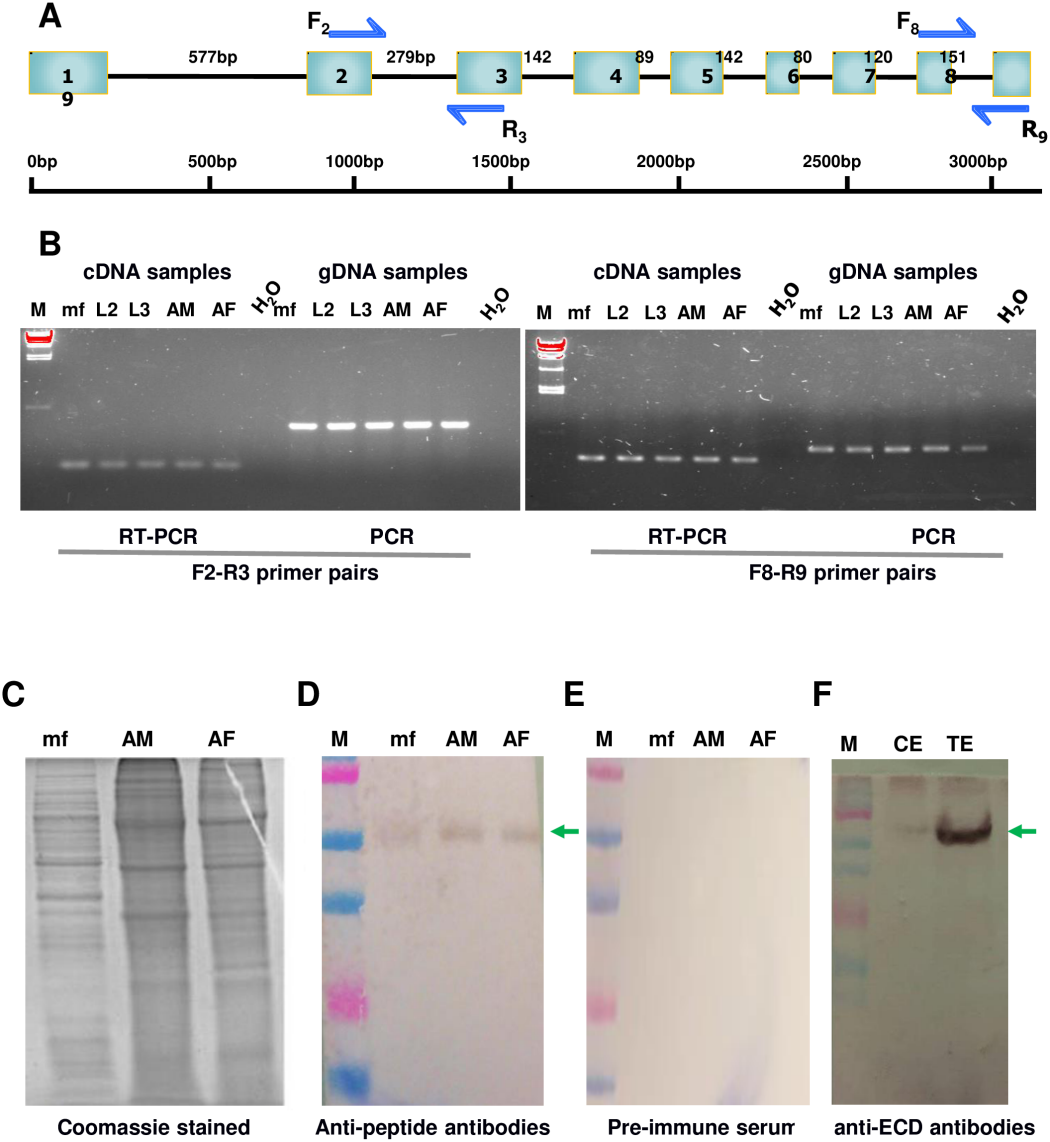
*Ov58GPCR* is transcriptionally and translationally activated in larva and adult parasite stages. (A) Two sets of primers targeting specific regions of *Ov58GPCR* across adjacent introns **were** used for stage-specific detection of *Ov58GPCR* in both genomic DNA and total cDNA preparations. (B) cDNA and genomic DNA of *O*. *volvulus* Mf, L2, L3, adult male (AM) and adult female (AF) parasitic stages were prepared and analysed. The first primer pair F2-R3 produced amplicons of 390 bp (from genomic DNA) and 111 bp (from cDNA) as predicted. The second primer set, F8-R9 specifically amplified a 338 bp product from genomic DNA and a 187 bp product from cDNA as predicted. The amplicons were resolved, ethidium bromide-stained and UV-visualized on agarose gels. (C) SDS-PAGE analysis and Coomassie staining of worm total protein extracts. (D-E) Western blot analysis of Ov58GPCR (green arrow) expression in *O*. *volvulus* extracts. (F) Western blot monitoring of Ov58GPCR (green arrow) in cuticular protein extract. mf: microfilaria extract, AM: adult male extract, AF: adult female extract, L2 and L3: parasite larval stages, CE: cuticular surface extract, TE: total extract.

An optimal diagnostic antigen should ideally be expressed throughout the parasite life cycle. To investigate stage specific expression, computer analyses were performed to design two independent primer pairs spanning exon/intron boundaries as shown in Fig 7A. Total RNA and genomic DNA (gDNA) samples were extracted as described previously by Ngale et al, [28]. The expression of Ov58GPCR was investigated in these samples using RT-PCR; PCR analysis on gDNA from the corresponding parasitic stages was used as control. Results obtained show that Ov58GPCR is transcriptionally expressed in all the tested *O*. *volvulus* larvae and adult worm stages. The PCR signals from gDNA samples correlated with the size of the intron flanked by the exons, which contained the primer binding sites (Fig 7B), showing in both cases different electrophoretic mobilities compared to the RT-PCR bands.

We proceeded to analyze protein expression of Ov58GPCR in microfilariae and adult protein extracts by western blot. The quality of the samples was first monitored by SDS-PAGE and Coomassie blue staining (Fig 7C). Resolved extracts transferred on blots were visualised using both the anti-peptide P4 and anti-ECD antibodies as well as the rabbit pre-immune sera as negative controls. Results obtained pointed at a single specific band at the expected size with the antibodies of interest (Fig 7D) while the pre-immune serum showed no signal (Fig 7E), suggesting the specificity of the bands of interest detected on the larvae and adult extracts.

Finally, we further addressed the authenticity of the unique bands visualised on Fig 7D. To this end, a cuticular surface protein extract was prepared from adult *O*. *volvulus* worm and characterized by western blot analysis. Consistent with the fact that the Ov58GPCR clone was initially isolated from a λgt-11 expression library by virtue of its reaction with a rabbit antibody against cuticular surface extract [82], the anti-ECD antibody detected a single band in the cuticular surface extract at the expected size (Fig 7F), suggesting that our antibody of interest is targeting the endogenous Ov58GPCR. Hence, we suggest that the generated anti-peptide and anti-ECD antibodies are specific for the protein in the worm extracts and could be used for further downstream applications, including antigen-capture test and assessment of RNAi-mediated knockdown of this receptor in our ongoing loss-of-function experiments.

## Discussion

In this communication, computational and experimental approaches were employed to assess Ov58GPCR, a G-protein coupled receptor of *Onchocerca volvulus*, as a target antigen useful for the engineering of molecular tools required to supplement current tools in monitoring progress made towards onchocerciasis elimination in communities where this disease is endemic. Its possible use is supported by the finding that synthetic peptides from the extracellular domain (ECD) as well as the recombinant Ov58GPCR-ECD polypeptide significantly discriminated serum samples from actively-infected subjects and chemotherapy-treated persons based on either antigen-specific IgG or IgE. Our study also suggests a degree of species-specificity as it showed a significantly differential response when sera from individuals infected with *O*. *volvulus* were compared to those infected with *Loa loa*, the infective agent of a closely related human filariasis. This result however has to be confirmed using sera from those infected with other parasitic nematodes to further assess the specificity.

We do acknowledge the need for a more rigorous characterisation of the serum samples used in studies to come. This includes the need to screen the different participants recruited for infection with other filarial nematodes as these nematodes could elicit cross-reactive responses to Ov58GPCR. This also includes the need to assess the cross-reactivity of our antibodies with extracts from related nematode species. We also acknowledge the putative limitation of microfilaridermia (as assessed by a skin snip) as a determinant for disease status especially among the control groups. As this technique is poorly sensitive (although still one of the only widely available) it could generate false negatives. The use of molecular diagnostic tools as the O-5S qPCR, O-150 qPCR [83] or the Ov16 test should be done in the future to confirm the skin snip results.

### In-silico functional structures of Ov58GPCR

Computational investigations focusing on Ov58GPCR revealed several structural features some of which correlate with previously reported experimental findings. With an estimated molecular weight of 58 kDa, Pfam analyses detected in this protein a Rhodopsin-like GPCR transmembrane domain. This is further supported by MEMSATSVM and TOPCONS servers whose results indicate the presence of 7 transmembrane helices and the extracellular N- and cytosolic C- termini [84] characteristic of GPCRs. Additionally, Ov58GPCR may harbour aspartate-glutamate racemase activities as Prosite [85] detected at positions 243–251 a potential racemase signature. Aspartate racemase and glutamate racemase are two evolutionary related bacterial enzymes which seem not to require a cofactor for their activity [86]. Glutamate racemase interconverts L-glutamate into D-glutamate and is required for the biosynthesis of peptidoglycan [87]. The enzyme is essential for growth across the bacterial kingdom with conserved overall topology and active site architecture [88]. Further investigations to ascertain the functionality of this motif whose presence in a eukaryotic protein is uncommon are therefore required since this enzyme has recently been suggested as a target in drug discovery efforts [88]. The protein also has relatively high percentage of Serine residues (8.7%) which could serve as sites for phosphorylation. Almost all G-protein coupled receptors (GPCRs) are regulated by phosphorylation and this posttranslational modification is a key event in determining the signalling properties of members of this receptor super-family [89]. Six of the eight conserved Cys residues in the protein and related homologues are located in the ECD where they could contribute to a conserved 3D structure required for a functional ligand binding pocket. In support of this possibility, the *C*. *elegans* orthologue of Ov58GPCR is reported to be affected by Sirolimus. Moreover, this *C*. *elegans* gene orthologue is predicted to be involved in chemotaxis and thermotaxis [90]. These data suggest future avenues of research for the functional characterization of the *O*. *volvulus* molecule.

### Diagnostic potentials of Ov58GPCR

The WHO’s ambitious goal of eliminating onchocerciasis in 80% of African countries by 2025 [91] will not be achieved without the development of reliable tools to certify disease elimination and subsequent post-elimination surveillance. Despite numerous attempts to develop simple tests, the Ov16 ELISA is the only immunodiagnostic test used to certify the interruption of disease transmission. It has only recently become available and it does not always test positive with subjects patently suffering from onchocerciasis [25,92]. In the midst of several approaches one key limiting factor to designing such tools is the shortage of knowledge on molecules potentially released into the blood stream such as the cuticular surface proteins shed as a result of parasite molting. This is probably the case of Ov58GPCR reported in this study. The two main requirements for an optimal diagnostic antigen are (i) its ability to discriminate between infected and healthy individuals, leading therefore to its capacity to differentiate past/treated and active infections, and (ii) its potential to specifically recognize/detect a target parasite species, a very challenging characteristic, given the high sequence identity amongst genes from related nematodes. Our investigations using synthetic peptides or peptide cocktails from Ov58GPCR-ECD as well as the recombinant ECD led to results that match these main requirements. Synthetic peptides have been used in the sero-diagnosis of many infectious disease with good performance [32–34] with their main advantage being the potential for reduced cross-reactivity often observed with recombinant and native antigens [93]. The Ov58GPCR peptides and peptide cocktails in this preliminary study were all able to discriminate between actively-infected onchocerciasis subjects and treated/non-infected individuals although to different extents. Peptide and peptide cocktails are therefore promising diagnostic candidates and need to be further evaluated. Antigen cocktails are generally reported to be more appropriate in sero-diagnostic and sero-surveillance studies since they have the capacity to achieve broad population coverage unlike single epitopes [94]. For this study, although single extracellular peptides were able to detect active infections, the intensity of the detected IgG response signal was amplified with peptide cocktails, as well as their ability to discriminate ongoing infections and treated individuals. Peptides cocktails P1-3, P1-2-4 and P1-3-4 are proposed for further analyses based on their diagnostic parameters (Table 2). Unlike the peptide cocktails which demonstrated a consistent pattern with the OVS, ITS and HES groups with significantly higher responses in the infected subjects, the purified ECD showed more variability within the tested groups and was not able to discriminate between OVS and ITS for total IgG. We argue that other epitopes in the ECD could generate cross-reaction, thereby neutralizing its ability to discriminate between the OVS and ITS samples. The peptides generally showed lower conservation than the entire protein and this could possibly be the reason for their ability to discriminate between the OVS and ITS groups. ECD was however able to differentiate them on the basis of antigen-specific IgE responses (Fig 5). This suggests ECD would be less reliable for assessing ivermectin efficiency on the basis of IgG detection, but this could be done on the basis of IgE responses.

On the other hand, although Ov58GPCR shows much conservation (Fig 1C), ECD-based ELISA discriminated between the LLS and the OVS groups while monitoring total IgG. This discrimination is important since co-infection with *Loa loa* is interfering with onchocerciasis control. The significant recognition of the ECD that the HES group displayed (Fig 5B), suggests possible cross-reaction with other unknown active or past pathologies faced by the Rwandan population. Also, one of the samples from the HES groups elicited a response that was markedly different from the others. This may be a result of an active or past *Onchocerca volvulus* infection since these people were not tested for onchocerciasis or cross-reactions with other nematode infections.

In addition, IgG subclass response comparisons between the ENS and OVS groups did not reveal any significant differences (data not shown). Since the produced anti-P4 antibodies are mono-specific, the generation of a polyclonal antibody was desired since it is reported that only subgroups of infected populations generally respond to single epitopes.

The marked fall in IgE levels in the ITS group (Fig 5B) is consistent with the concept of high IgE titres in active onchocerciasis infections [95,96]. It is well known that helminth parasites have immunomodulatory effects on their hosts [97]. They characteristically cause a skew towards T_H_2 immunity, stimulating immunoregulatory cell populations, such as regulatory T cells (T_regs_) and alternatively activated macrophages (AAMs), while simultaneously inhibiting T_H_1 and T_H_17. This immunomodulation enables evasion from host immunity for maturation, and long-term survival of the parasite in what has been termed a ‘modified T_H_2’ environment [81]. Additionally, they induce eosinophilia and extensive IgE release [80]. It has been reported that drug treatment to clear the worms reverses the immunoregulatory effects, terminating the state of active suppression maintained by the parasite [81]. In the context of a drug program evaluation, data obtained suggests that IgG subclasses (particularly IgG_4_) responses to Ov58GPCR-ECD could be useful in real-time monitoring and evaluation of control program successes while IgE may be more applicable in post-elimination surveillance. The declines in antigen-specific IgG subclasses and IgE levels with increased ivermectin treatment length corroborates the concept of decreasing antigenic stimulation following chemotherapy [75]. Similar results were obtained by Soboslay *et al*., [98] measuring total IgG and IgG subclasses in onchocerciasis following ivermectin administration over an 84 month period and by Atmadja *et al*., [99] in *Brugia malayi*-infected individuals following diethylcarbamazine chemotherapy over a 2.5-year period. It was however observed that following chemotherapy, there was maintenance of a sustained antigen-IgE response before the levels eventually felt and levelled-off with continuous ivermectin treatment. The sustenance of circulating antigen-specific IgE following chemotherapy may be a result of increased IL-4 levels as reported by Gillespie at al., [75] which could lead to immunopotentiation at the initiation of chemotherapy. The initially augmented immune response eventually falls with repeated chemotherapy. Though the levels of both antigen-specific IgG and IgE declined in our case, the marked decline in IgE following ivermectin treatment could be a homeostatic response of the body to maintain low, maintenance levels of parasite-specific IgE in the absence of infection. Remaining high IgG levels in the endemic non symptomatic group agrees with the work of Ghogomu *et al*., [27] who reported that sera from both infected and endemic non symptomatic reacted with Ov-47 in immunoblot assays. The maintenance of elevated IgE/IgG levels in the LLS group (Fig 5) could mainly be a result of the conservation of the protein in both *L*.*loa* and *O*. *volvulus* as seen from the phylogenetic tree (Fig 1C).

Since the gene was transcriptionally active in all the parasite stages, and the protein detected in the microfilaria and adult stages, it can be suggested that the protein plays an important role in the life of the parasite. RNAi and immunohistochemistry are under way and may give more insight into the function of this protein.

## Conclusion

This work provides a proof-of-concept for the use of the extra-cellular domain of Ov58GPCR as well as its peptide cocktails in monitoring the efficacy of ivermectin chemotherapeutic programs for onchocerciasis and for the detection of new infections based on antigen-specific IgE responses. The recombinant extracellular domain (Ov58GPCR-ECD) or cocktails of peptides from this domain discriminated significantly sera from treated and actively infected persons. The gene encoding this antigen is expressed in both larvae and adult worms. Polyclonal antibodies against its extracellular domain or a peptide from this domain detected a single band in the parasite total protein extract, as well as in the adult cuticular extract, suggesting its potential presence in blood stream of infected individuals (possibly after release upon parasite molting). We conclude therefore that Ov58GPCR is a candidate antigen that deserves to be further studied in order to design serological assays required to assess the progress made by onchocerciasis control programs. As reliable and field applicable molecular test to monitor *Onchocerca* infection is still missing, we believe that such a test should target several antigens at a time. We believe that Ov58GPCR would be a significant contributor to this collection of antigens.

## Supporting information

S1 Fig Ov58GPCR is a 7-transmembrane protein conserved in related nematodes. (TIF)

S2 Fig Ov58GPCR harbours immunogenic epitopes in its extracellular domain. (TIF)

S3 Fig Increased antigen-specific antibody responses to Ov58GPCR peptide cocktails are associated with onchocerciasis infection. (TIF)

S4 Fig The recombinant extra-cellular domain of Ov58GPCR (Ov58GPCR-ECD) can be found in monomeric and dimeric forms. (TIF)

S5 Fig Exposure of host to parasite transmission leads to increased Ov58GPCR-ECD-specific IgG but not IgE response following treatment. (TIF)

S6 Fig Increased ivermectin treatment length is associated with decline in IgG subclasses and total IgE responses to Ov58GPCR-ECD. (TIF)

S7 Fig *Ov58GPCR* is transcriptionally and translationally activated in larva and adult parasite stages. (TIF)

S1 Table Physicochemical properties of predicted B-epitopes. (PDF)

S2 Table Diagnostic accuracy parameters for Ov58GPCR peptides and peptide cocktails. (PDF)

S3 Table Raw ELISA data. (XLSX)
