# Supplementary figures and images for: Prediction and validation of the structural features of Ov58GPCR, an immunogenic determinant of Onchocerca volvulus
Source: PLoS One. 2018 Sep 26;13(9):e0202915. doi: 10.1371/journal.pone.0202915 (PMC6157839; doi:10.1371/journal.pone.0202915)

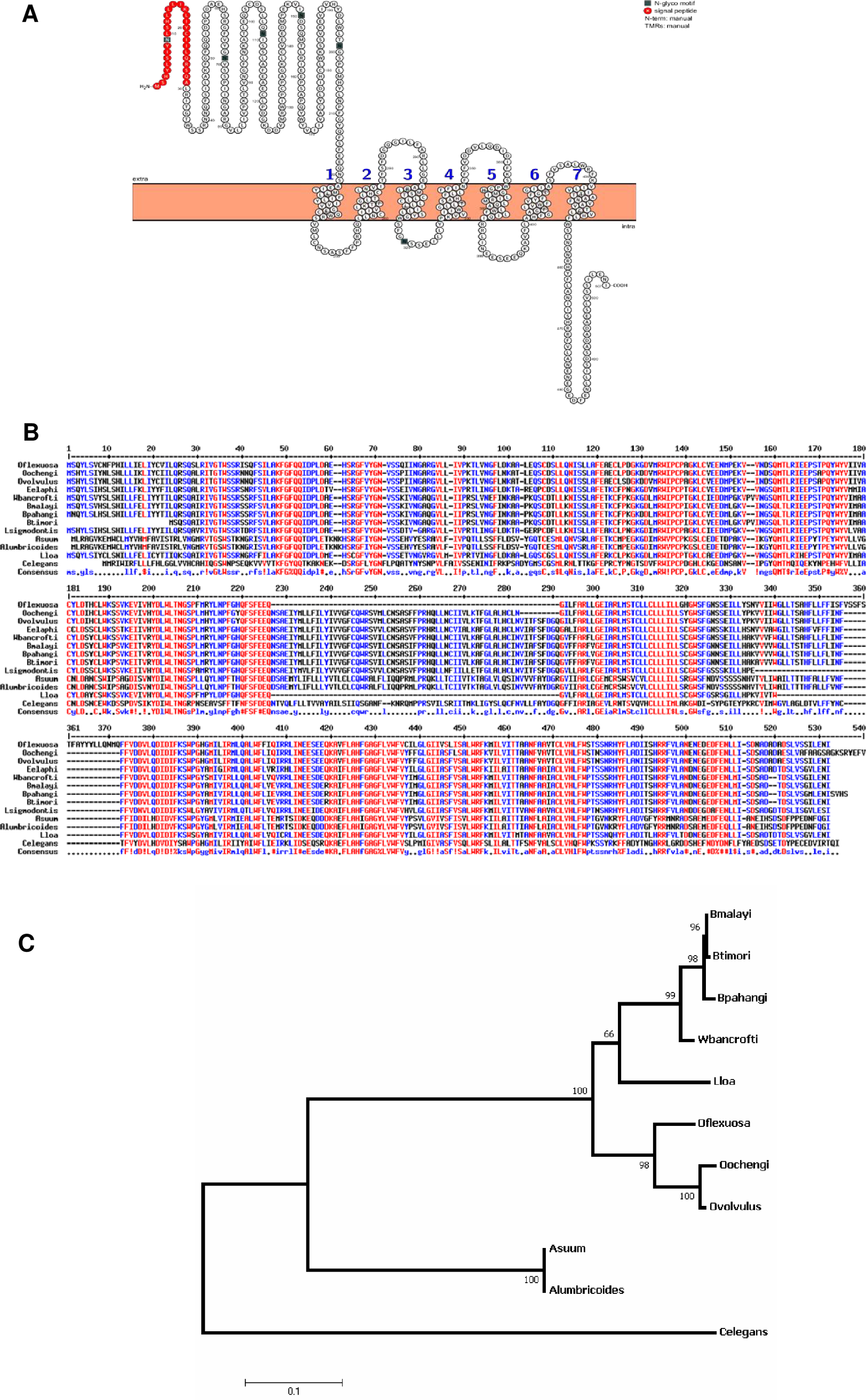

Supplement: S1 Fig — (TIF) [file pone.0202915.s001.tif]

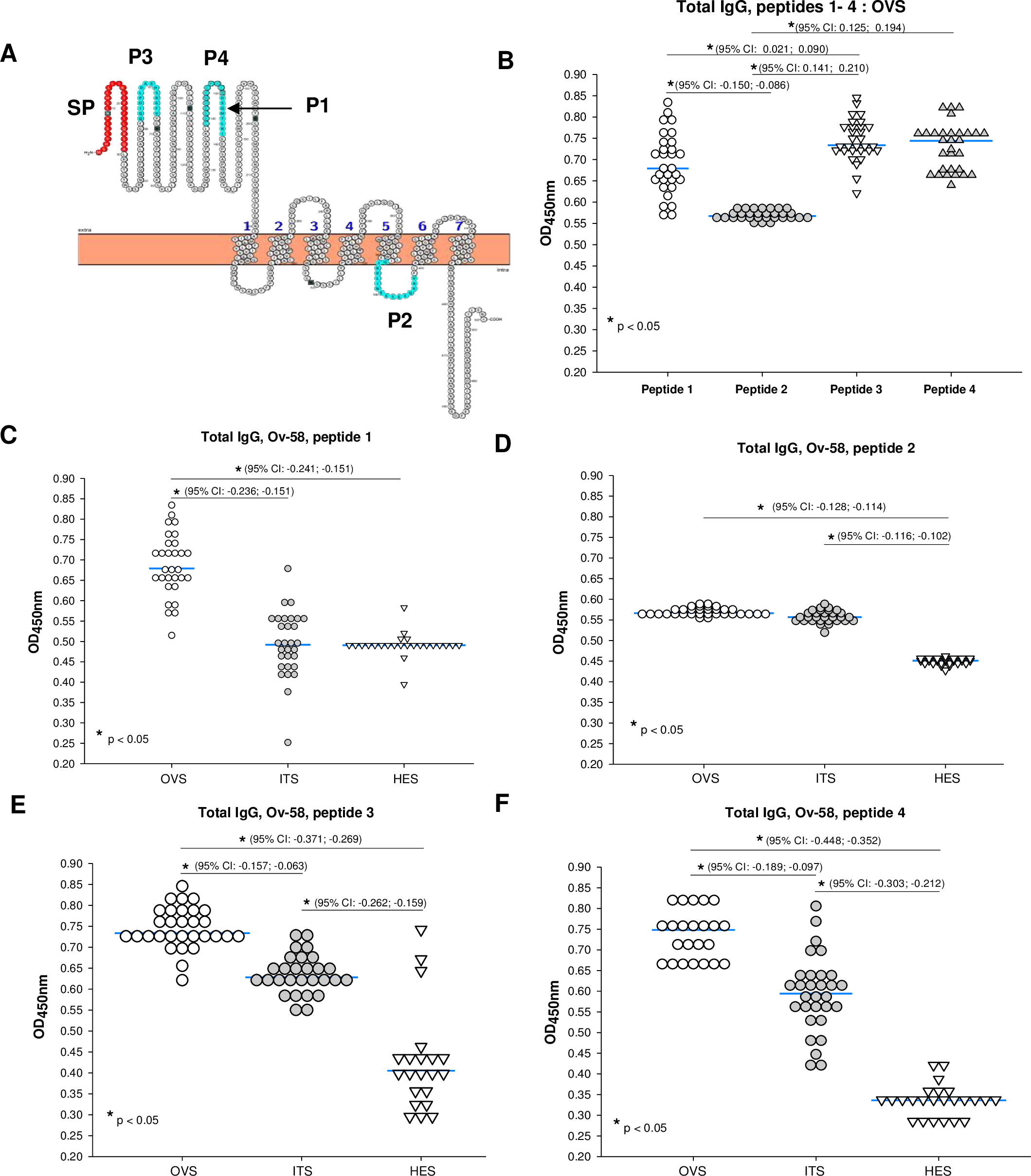

Supplement: S2 Fig — (TIF) [file pone.0202915.s002.tif]

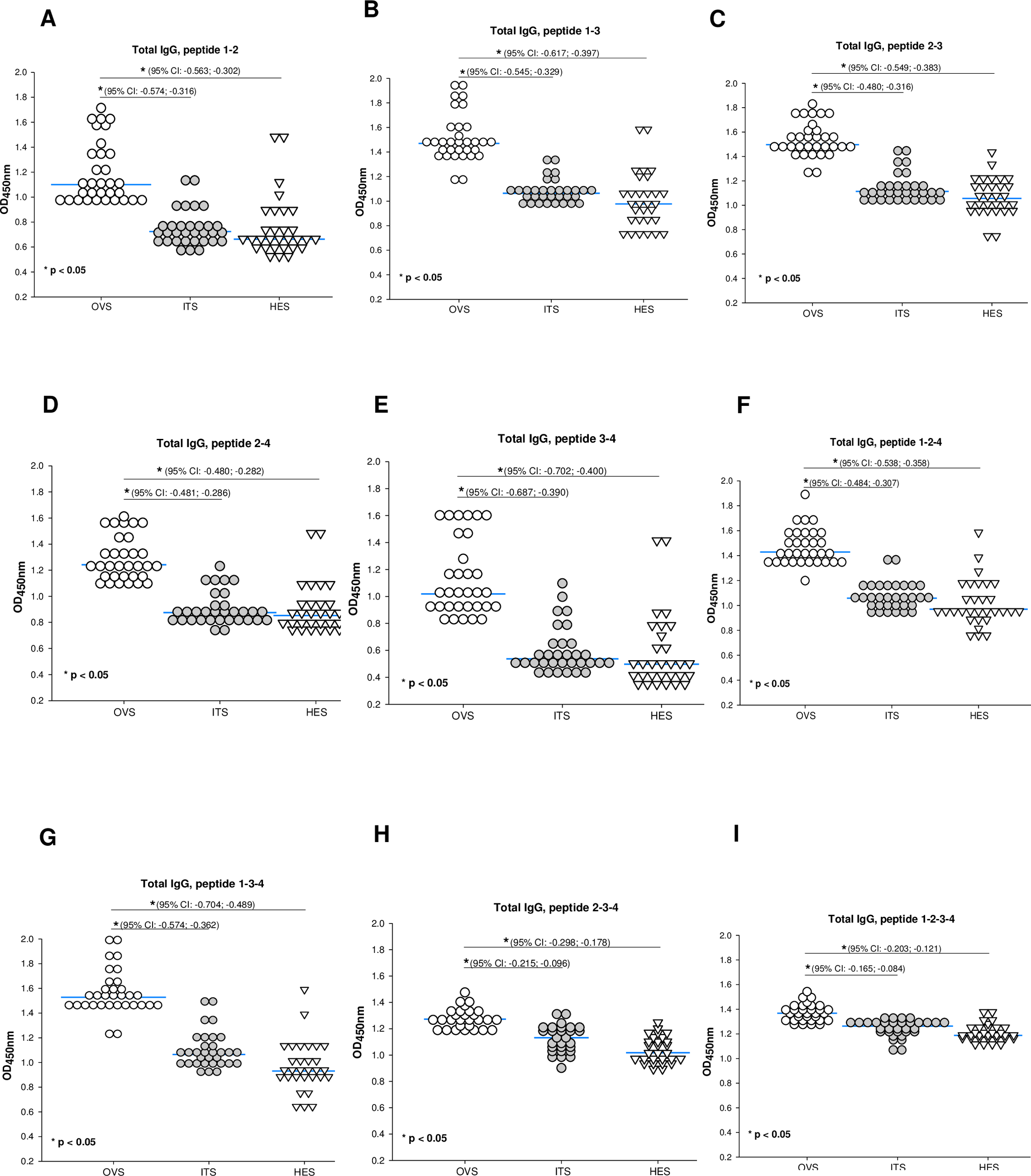

Supplement: S3 Fig — (TIF) [file pone.0202915.s003.tif]

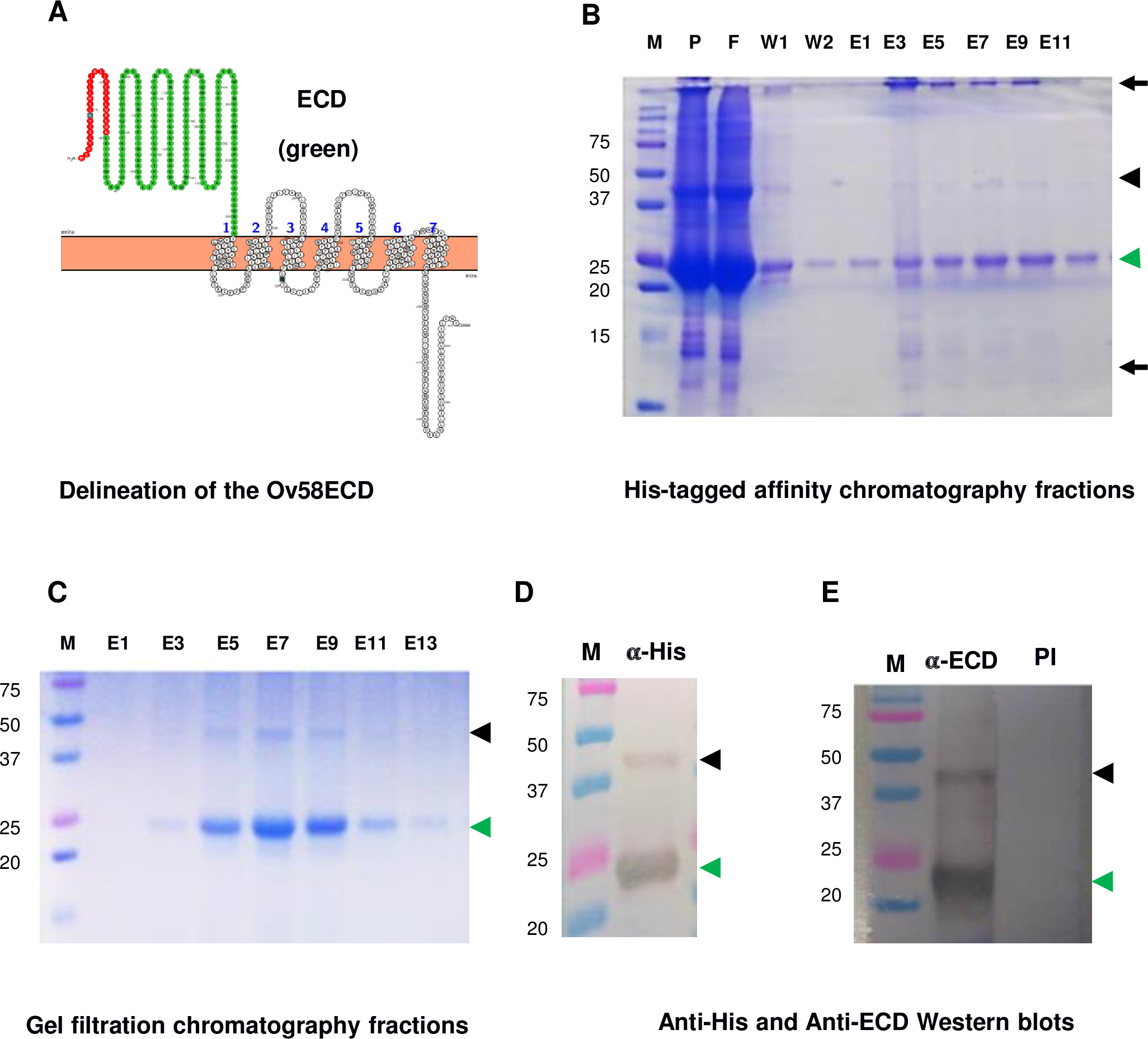

Supplement: S4 Fig — (TIF) [file pone.0202915.s004.tif]

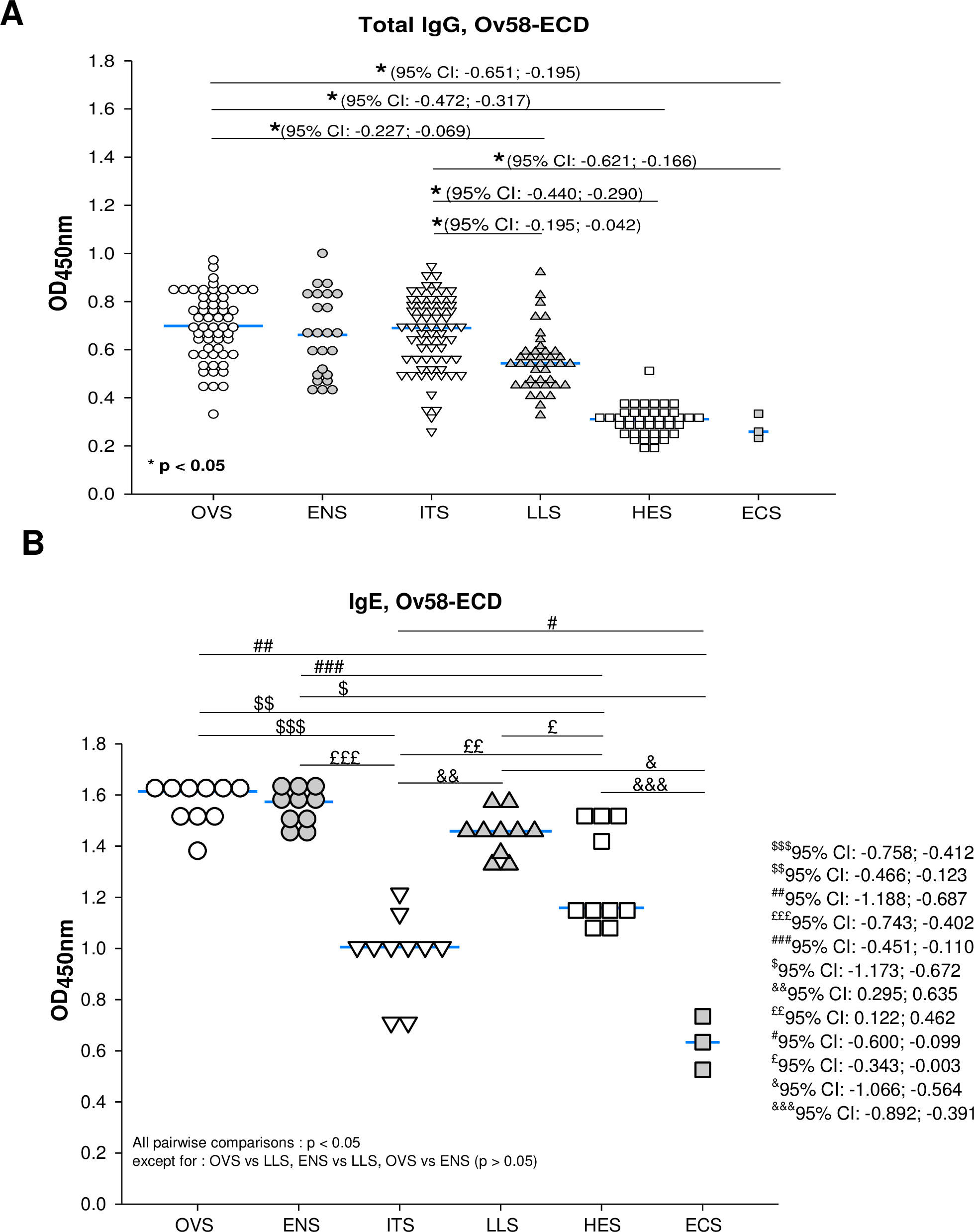

Supplement: S5 Fig — (TIF) [file pone.0202915.s005.tif]

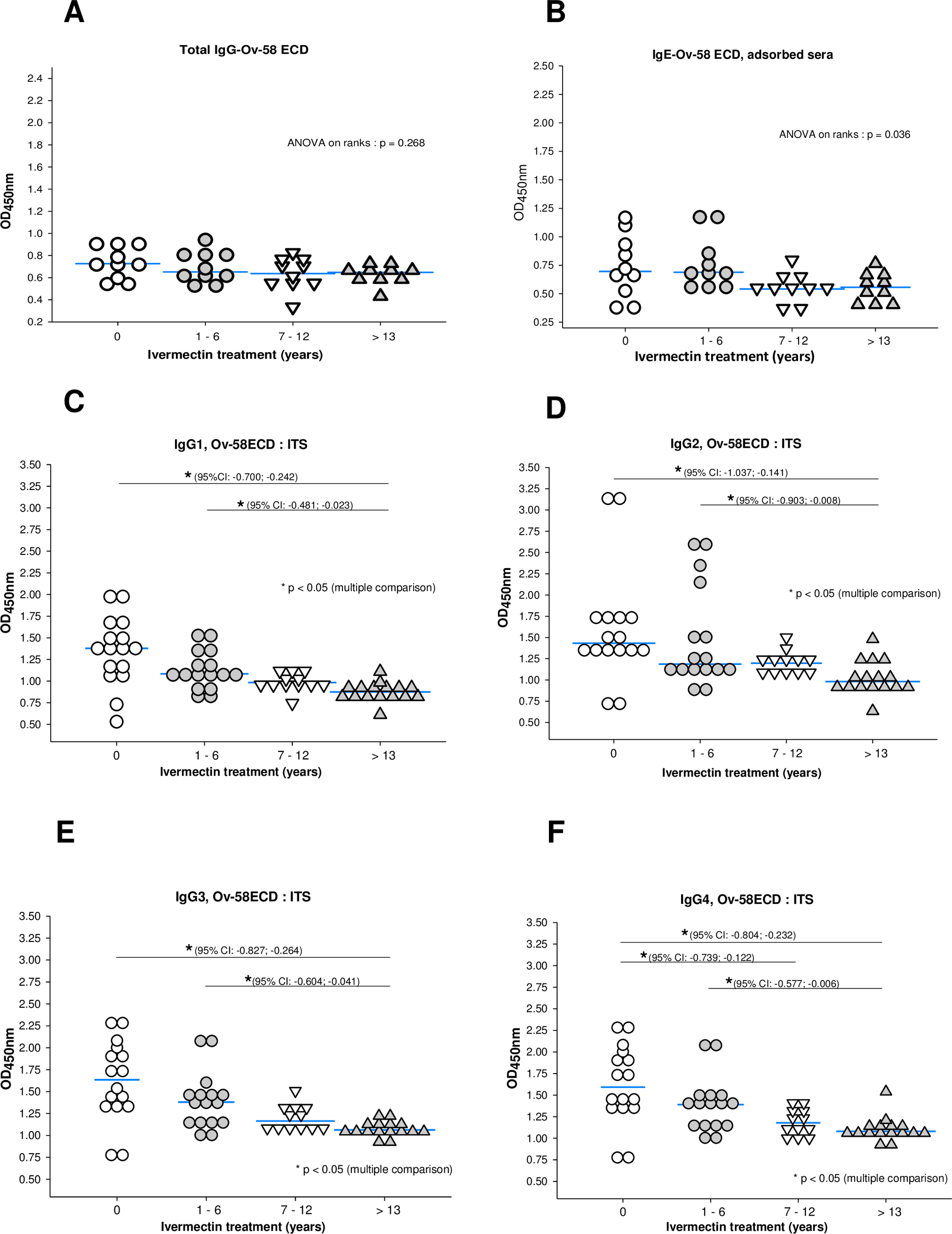

Supplement: S6 Fig — (TIF) [file pone.0202915.s006.tif]

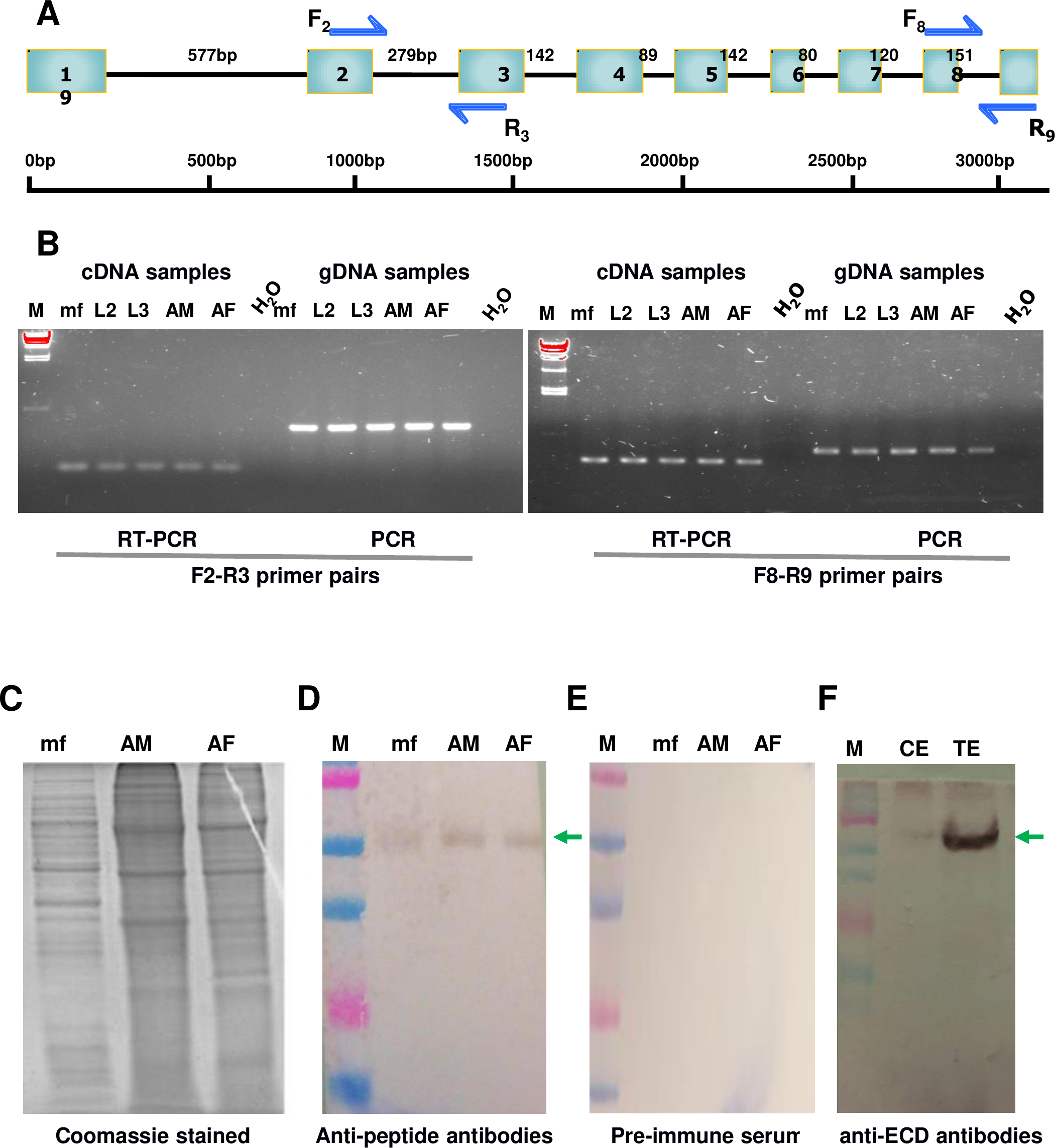

Supplement: S7 Fig — (TIF) [file pone.0202915.s007.tif]
