# Supplementary material for: Prediction and validation of the structural features of Ov58GPCR, an immunogenic determinant of Onchocerca volvulus
Source: PLoS One. 2018 Sep 26;13(9):e0202915. doi: 10.1371/journal.pone.0202915 (PMC6157839; doi:10.1371/journal.pone.0202915)
Supplement: S1 Table — (PDF) [file pone.0202915.s008.pdf]

Peptide	Mw (Da)	Secondary structure type	pI	AA[b]	GV	AI
**P1: CVEEDMPEKVINDSQMTLRI**	**2350.70**	**Coil/strand**	**4.18**	**139 - 158**	**-0.425**	**87.5**
**P2: IRRLINEESEEQKAV**	**1814.03**	**Helix**	**4.95**	**383 - 398**	**-1.060**	**104.0**
**P3: QQIDPLDAEHSRGFV**	**1711.85**	**Coil**	**4.54**	**52 - 66**	**-0.727**	**78.0**
**P4: CVEEDMPEKVIN**	**1405.60**	**Coil**	**4.0**	**139 - 150**	**-0.475**	**80.8**
