# Supplementary material for: Prediction and validation of the structural features of Ov58GPCR, an immunogenic determinant of Onchocerca volvulus
Source: PLoS One. 2018 Sep 26;13(9):e0202915. doi: 10.1371/journal.pone.0202915 (PMC6157839; doi:10.1371/journal.pone.0202915)
Supplement: S2 Table — (PDF) [file pone.0202915.s009.pdf]

Peptide	Area Under the ROC Curve (AUC)	Standard Error	95% CI for ROC AUC	p value	Selected cutoff	Sensitivity for selected cutoff and 95% CI		Specificity for selected cutoff and 95% CI		Positive likelihood ratio and 95% CI for selected cutoff		Negative likelihood ratio and 95% CI for selected cutoff	
**Peptide 1**	0.977	0.0135	[0.918 - 0.997]	<0.0001	0.5599	96.67	[82.8 - 99.9]	92.59	[82.1 - 97.9]	13.05	[5.1 - 33.6]	0.036	[0.005 - 0.2]
**Peptide 2**	0.858	0.0399	[0.766 - 0.924]	<0.0001	0.5581	93.75	[79.2 - 99.2]	77.78	[64.4 - 88.0]	4.22	[2.5 - 7.0]	0.08	[0.02 - 0.3]
**Peptide 3**	0.952	0.0222	[0.880 - 0.988]	<0.0001	0.687	93.33	[77.9 - 99.2]	89.8	[77.8 - 96.6]	9.15	[4.0 - 21.1]	0.074	[0.02 - 0.3]
**Peptide 4**	0.951	0.0241	[0.876 - 0.987]	<0.0001	0.6535	100	[85.2 - 100.0]	90.74	[79.7 - 96.9]	10.8	[4.7 - 24.9]	0	-
**Peptide 1-2**	0.939	0.0256	[0.870 - 0.978]	<0.0001	0.946	100	[89.1 - 100.0]	90.32	[80.1 - 96.4]	10.33	[4.8 - 22.1]	0	-
**Peptide 1-3**	0.967	0.0181	[0.908 - 0.993]	<0.0001	13.347	93.75	[79.2 - 99.2]	96.77	[88.8 - 99.6]	29.06	[7.4 - 114.0]	0.065	[0.02 - 0.2]
**Peptide 2-3**	0.987	0.0074	[0.939 - 0.999]	<0.0001	12.686	93.75	[79.2 - 99.2]	90.32	[80.1 - 96.4]	9.69	[4.5 - 20.8]	0.069	[0.02 - 0.3]
**Peptide 2-4**	0.951	0.0217	[0.886 - 0.985]	<0.0001	1.095	96.87	[83.8 - 99.9]	88.71	[78.1 - 95.3]	8.58	[4.3 - 17.3]	0.035	[0.005 - 0.2]
**Peptide 3-4**	0.95	0.0218	[0.884 - 0.984]	<0.0001	0.7893	100	[89.1 - 100.0]	87.1	[76.1 - 94.3]	7.75	[4.1 - 14.8]	0	-
**Peptide 1-2-4**	0.97	0.0166	[0.912 - 0.994]	<0.0001	11.941	100	[89.1 - 100.0]	91.94	[82.2 - 97.3]	12.4	[5.4 - 28.7]	0	-
**Peptide 1-3-4**	0.972	0.0155	[0.915 - 0.995]	<0.0001	12.237	100	[89.1 - 100.0]	90.32	[80.1 - 96.4]	10.33	[4.8 - 22.1]	0	-
**Peptide 2-3-4**	0.93	0.026	[0.853 - 0.974]	<0.0001	11.743	100	[87.7 - 100.0]	76.79	[63.6 - 87.0]	4.31	[2.7 - 6.9]	0	-
**Peptide 1-2-3-4**	0.929	0.0246	[0.858 - 0.972]	<0.0001	13.012	84.37	[67.2 - 94.7]	87.5	[76.8 - 94.4]	6.75	[3.5 - 13.1]	0.18	[0.08 - 0.4]
